# Supplementary figures and images for: Associations between indoor temperature, self-rated health and socioeconomic position in a cross-sectional study of adults in England
Source: BMJ Open. 2021 Feb 23;11(2):e038500. doi: 10.1136/bmjopen-2020-038500 (PMC7907859; doi:10.1136/bmjopen-2020-038500)

**Figure S1. Flow diagram showing selection of data into analytical sample**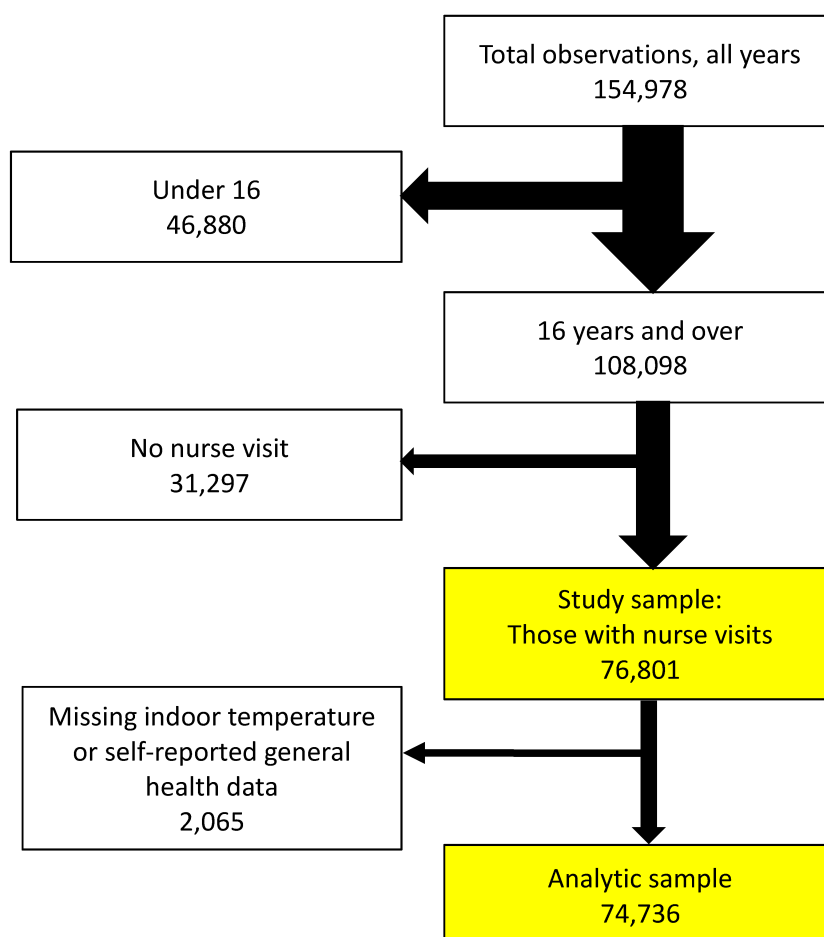

Supplement: Supplementary data [file bmjopen-2020-038500supp001.pdf]
