# Supplementary material for: Associations between indoor temperature, self-rated health and socioeconomic position in a cross-sectional study of adults in England
Source: BMJ Open. 2021 Feb 23;11(2):e038500. doi: 10.1136/bmjopen-2020-038500 (PMC7907859; doi:10.1136/bmjopen-2020-038500)
Supplement: Supplementary data [file bmjopen-2020-038500supp002.pdf]

Supplementary material

Table S1. Characteristics of participants

Variable	Category	Analytical sample[a] (N=74,736)		Excluded from analysis[b] (N=2065)		Study Sample[c] (N=76,801)	
		N*	%	N	%	N	%
Gender[d]	Men	33,526	45%	533	26%	34,059	44%
	Women	41,210	55%	1,532	74%	42,742	56%
Age group[d]	16-24	6,593	9%	373	18%	6,966	9%
	25-34	9,589	13%	639	31%	10,228	13%
	35-44	13,145	18%	375	18%	13,520	18%
	45-54	12,929	17%	179	9%	13,108	17%
	55-64	12,753	17%	161	8%	12,914	17%
	65-74	11,315	15%	162	8%	11,477	15%
	75+	8,412	11%	176	9%	8,588	11%
NS-SEC5[d]	Managerial/ professional	25,297	34%	557	27%	25,854	34%
	Intermediate Occupation	10,354	14%	234	11%	10,588	14%
	Small employers	6,514	9%	91	4%	6,605	9%
	Lower supervisory	6,087	8%	96	5%	6,183	8%
	Semi-routine	23,314	31%	511	25%	23,825	31%
	Missing data	*3,170*	*4%*	132	6%	*3,302*	*4%*
Education[d]	Degree level or higher	15,400	21%	476	23%	15,876	21%
	Qualification below degree	40,233	54%	992	48%	41,225	54%
	No qualification	19,103	26%	515	25%	19,618	26%
	Missing data	*0*	*0%*	*82*	*4%*	*82*	*0.1%*
Income quintile[d]	Lowest	10,861	15%	420	20%	11,281	15%
	2nd lowest	12,115	16%	302	15%	12,417	16%
	3rd	13,190	18%	260	13%	13,450	18%
	4th	13,537	18%	265	13%	13,802	18%
	Highest	13,291	18%	321	16%	13,612	18%
	Missing data	*11,742*	*16%*	461	22%	*12,203*	*16%*
Tenure[d]	Owner-occupied	55,159	74%	1,076	52%	56,235	73%
	Privately rented	6,476	9%	283	14%	6,759	9%
	Socially rented	11,347	15%	492	24%	11,839	15%
	Missing data	*1,754*	*2%*	214	10%	*1,968*	*3%*
Household size[d]	1	13,677	18%	284	14%	13,961	18%
	2	30,117	40%	712	34%	30,829	40%
	3	12,585	17%	540	26%	13,125	17%
	4	12,173	16%	323	16%	12,496	16%
	5+	6,184	8%	206	10%	6,390	8%

Tertile of regional mean outdoor temperature at month of visit[e]	Low	24,927	33%	653	32%	25,580	33%
	Medium	24,506	33%	752	35%	25,238	33%
	High	25,303	34%	680	33%	25,983	34%
General health[f]	Good	55,235	74%	1,567	76%	56,802	74%
	Poor	19,501	26%	498	24%	19,999	26%
Longstanding illness[d]	No	39,246	53%	1,269	61%	40,515	53%
	Yes	35,490	47%	781	38%	36,271	47%
Heart condition[d]	No	63,905	86%	1,838	89%	65,743	86%
	Yes	10,831	14%	212	10%	11,043	14%
Respiratory condition[g]	No	68,051	91%	1,869	91%	69,920	91%
	Yes	6,685	9%	181	9%	6,866	9%
Total		*74,736*	*97.3%*	*2,065*	*2.7%*	*76,801*	*100%*

[a] Analytical sample was all participants with a nurse visit and with information on both self-rated health and indoor temperature'.

[b] Excluded sample are those HSE participants who had a nurse visit (so are included in the study sample) but had missing data for indoor temperature and/or self-rated health

[c] Study sample: all those with a nurse visit

[d] X^2 test for difference between analytic sample and excluded participants: p<0.001

[e] X^2 test for difference between analytic sample and excluded participants: p=0.036

[f] X^2 test for difference between analytic sample and excluded participants: p=0.043

[g] X^2 test for difference between analytic sample and excluded participants: p=0.858
