# Supplementary material for: Associations between indoor temperature, self-rated health and socioeconomic position in a cross-sectional study of adults in England
Source: BMJ Open. 2021 Feb 23;11(2):e038500. doi: 10.1136/bmjopen-2020-038500 (PMC7907859; doi:10.1136/bmjopen-2020-038500)
Supplement: Author's manuscript [file bmjopen-2020-038500.draft_revisions.pdf]

BMJ Open

BMJ Open is committed to open peer review. As part of this commitment we make the peer review history of every article we publish publicly available.

When an article is published we post the peer reviewers' comments and the authors' responses online. We also post the versions of the paper that were used during peer review. These are the versions that the peer review comments apply to.

The versions of the paper that follow are the versions that were submitted during the peer review process. They are not the versions of record or the final published versions. They should not be cited or distributed as the published version of this manuscript.

BMJ Open is an open access journal and the full, final, typeset and author-corrected version of record of the manuscript is available on our site with no access controls, subscription charges or pay-per-view fees (http://bmjopen.bmj.com).

If you have any questions on BMJ Open's open peer review process please email info.bmjopen@bmj.com

BMJ Open

Associations between indoor temperature, self-rated health and socio-economic position in a cross-sectional study of adults in England

Journal:	*BMJ Open*
Manuscript ID	bmjopen-2020-038500
Article Type:	Original research
Date Submitted by the Author:	27-Apr-2020

Complete List of Authors:	Sutton-Klein, Joanna; UCL, Epidemiology and public health<br>Moody, Alison; UCL, Epidemiology and public health<br>Hamilton, Ian; University College London, UCL Energy Institute<br>Mindell, Jennifer; UCL (University College London), Epidemiology & Public Health

Keywords:	EPIDEMIOLOGY, PUBLIC HEALTH, SOCIAL MEDICINE

SCHOLARONE™
Manuscripts

I, the Submitting Author has the right to grant and does grant on behalf of all authors of the Work (as defined in the below author licence), an exclusive licence and/or a non-exclusive licence for contributions from authors who are: i) UK Crown employees; ii) where BMJ has agreed a CC-BY licence shall apply, and/or iii) in accordance with the terms applicable for US Federal Government officers or employees acting as part of their official duties; on a worldwide, perpetual, irrevocable, royalty-free basis to BMJ Publishing Group Ltd ("BMJ") its licensees and where the relevant Journal is co-owned by BMJ to the co-owners of the Journal, to publish the Work in this journal and any other BMJ products and to exploit all rights, as set out in our licence.

The Submitting Author accepts and understands that any supply made under these terms is made by BMJ to the Submitting Author unless you are acting as an employee on behalf of your employer or a postgraduate student of an affiliated institution which is paying any applicable article publishing charge ("APC") for Open Access articles. Where the Submitting Author wishes to make the Work available on an Open Access basis (and intends to pay the relevant APC), the terms of reuse of such Open Access shall be governed by a Creative Commons licence – details of these licences and which Creative Commons licence will apply to this Work are set out in our licence referred to above.

Other than as permitted in any relevant BMJ Author's Self Archiving Policies, I confirm this Work has not been accepted for publication elsewhere, is not being considered for publication elsewhere and does not duplicate material already published. I confirm all authors consent to publication of this Work and authorise the granting of this licence.

For peer review only

Associations between indoor temperature, self-rated health and socio-economic position in a cross-sectional study of adults in England

Authors:

Dr Joanna Sutton-Klein, MB ChB, MSc, Health and Social Surveys Research Group, Research Dept of Epidemiology and Public Health, UCL, 1-19 Torrington Place, London WC1E 6BT, UK. ORCID 0000-0002-7784-0986

Alison Moody, MSc, Health and Social Surveys Research Group, Research Dept of Epidemiology and Public Health, UCL, 1-19 Torrington Place, London WC1E 6BT, UK. ORCID 0000-0002-8199-4830

Dr Ian Hamilton, PhD, UCL Energy Institute, Bartlett Faculty of the Built Environment, UCL, 14 Upper Woburn Place, London, WC1H 0NN, UK. ORCID 0000-0003-2582-2361

Prof Jennifer S. Mindell, PhD, Health and Social Surveys Research Group, Research Dept of Epidemiology and Public Health, UCL, 1-19 Torrington Place, London WC1E 6BT, UK. ORCID 0000-0002-7604-6131

Corresponding author:

Dr Ian Hamilton, UCL Energy Institute, Bartlett Faculty of the Built Environment, UCL, Central House, 14 Upper Woburn Place, London, WC1H 0NN

Email: i.hamilton@ucl.ac.uk

Telephone: +44-20-3108-5982

Research report manuscript for *BMJ Open*

Word count: 2,830

Tables: 3 plus one supplementary table

Figures: 2 plus one supplementary figure

References: 36

Subject areas: Public health; Health policy

Journal keywords: Health status, Socio-economic inequalities, Temperature

Funding

AM & JM are funded by NHS Digital to work on the annual HSE. IH is funded by EPSRC [EP/R035288/1] to work on building energy efficiency. NHS Digital and EPSRC played no part in the planning, conduct, or interpretation of these analyses; the drafting of the manuscript; nor the decision to publish.

For peer review only - http://bmjopen.bmj.com/site/about/guidelines.xhtml

Abstract

Objective

Excess winter deaths (EWDs) are a major public health concern in England and Wales, with an average of 20,000 deaths per year since 2010. Feeling cold at home during winter is associated with reporting poor general health; cold and damp homes have greater prevalence in lower socioeconomic groups. This study evaluates the association between indoor temperature and general health and the extent to which this is affected by socioeconomic and household factors.

Design

Cross-sectional study.

Setting

England.

Participants

Secondary data of 74,736 individuals living in England that took part in the Health Survey for England (HSE) between 2003 and 2014. The HSE is an annual household survey which uses multilevel stratification to select a new, nationally-representative sample each year. The study sample comprised adults who had a nurse visit; the analytical sample was adults who had observations for indoor temperature and self-rated health.

Results

Using both logistic and linear regression models to examine indoor temperature and health status, adjusting for socio-economic and housing factors, the study finds an association between ill-health and higher indoor temperatures was found. Each one degree increase in indoor temperature was associated with a 1·7% (95% Confidence Interval (CI) 0·7%-2·6%) increase in the odds of poor health. After adjusting for income, education, employment type, household size and home ownership, the odds ratio (OR) of poor health for each degree temperature rise increased by 19%, to 1·02 (95%CI 1·01-1·03 P<0·01).

Conclusion

People with worse self-reported health had higher indoor temperatures after adjusting for household factors. Ill households may have chosen to maintain warmer environments or been advised to. However, other latent factors, such as housing type and energy performance could have an effect.

Article summary: Strengths and limitations of this study

- This study is based on data from 74,736 individuals, randomly sampled from the general population to be nationally-representative of the population in England.
- Temperature measurements were taken using a standard protocol across the 10 years.
- Analyses used individual-level data, not ecological comparisons.
- Indoor temperatures were measured only once per participant.
- Data are cross-sectional, so causality cannot be inferred.

For peer review only - http://bmjopen.bmj.com/site/about/guidelines.xhtml

Introduction

Short-term exposure to extreme hot and cold temperatures is known to be acutely harmful. For example, Analitis *et al.* compared the short-term effects of cold on mortality in 15 European cities, finding that a 1°C decrease in outdoor temperature was associated with a 1·35% increase in the daily number of total natural deaths.[1] A recent systematic review has shown the adverse health consequences of high indoor temperatures, above 26°C.[2] However, the long-term effects of exposure to different temperatures are not well understood. Much of the evidence on long-term exposure to more minor deviations in ambient temperature is overwhelmingly drawn from ecological studies.[3-5]

There were 49,410 excess winter deaths (EWDs) in England and Wales over the 2017-18 winter.[6] Although EWDs were significantly lower in 2018-19 (23,200, provisional figure),[6] the large inequalities in EWD index between European countries suggest a major public health problem.[7] Paradoxically, while several studies have found that cooler ambient temperatures are associated with higher mortality on a day-by-day analysis, European countries with colder winters were found to have lower excess winter deaths.[8] Studies have suggested that it is the variation in housing quality and the resulting indoor temperature differences which may instead be the drivers of these differences in health outcomes.[9, 10]

Few studies have directly addressed the association between indoor temperature and health in individuals, but a number have studied known correlates of indoor temperature such as damp, insulation, or perceived thermal comfort.[11, 12] These studies overwhelmingly found that proxies for cool indoor temperature were associated with worse health.

Several studies have compared the health of individuals before and after housing interventions. Upgraded insulation in New Zealand was associated with increased indoor temperature, and lower levels of poor general health (odds ratio, (OR) 0·50, 95% confidence interval (CI) 0·38-0·68).[13, 14] Furthermore, a large pan-European study found that perceived coldness at home was strongly associated with poor general health, with an OR of 2.6 (95% CI 2.1-3.1),[15] a result which was replicated in a study of English adults.[16] A limitation of using perceived thermal comfort as a proxy of indoor temperature is that its subjectivity makes it vulnerable to reverse causation.

Analysis of the 1991 survey of English houses found that the strongest predictors of low indoor temperatures were age of property, absence of central heating, cost of heating, small household size (i.e. fewer occupants), and low income.[17] A 2011 survey of English houses found similar importance of predictors of indoor temperature; that older properties were not the coldest; but that low energy performance ratings and low income households had the lowest temperatures.[18]

A systematic review by Public Health England concluded that living in cold temperatures (below 18°C) may increase the risk of adverse health consequences, particularly for premature mortality in vulnerable populations.[19] However, recommendations for minimum indoor temperature are based on limited evidence.[15] Research has shown that few households are keeping the recommended indoor temperatures (i.e. ≥18°C), particularly vulnerable households.[20] Recently, increasing poverty was associated with higher risk of poor housing within the UK.[21, 22] Analysis of Census longitudinal study data has shown a rise in the proportion of homes with central heating from 83% in 1991, to 93% in 2001 and 98% in 2011.[23] The English Housing Survey in 2016 found that prevalence of cavity or solid wall insulation, of double glazing, and of central heating had increased in England to

For peer review only - http://bmjopen.bmj.com/site/about/guidelines.xhtml

49%, 83%, and 92% of households respectively.[24] A study on government interventions found that the installation of heating and insulation was effective at increasing indoor temperatures.[25]

The aim of our study was to investigate the relationship between indoor temperature and general health, and to examine the extent to which socioeconomic and housing variables explain any association found. We hypothesised that: socioeconomic and housing factors would be associated with indoor temperature; poor indoor temperature control would be associated with worse general health; and the association between temperature and general health would be stronger with decreasing socioeconomic status.

Methods

Participants

The study sample was adults aged 16 years and over who took part in the nurse visit component of the Health Survey for England (HSE) between 2003 and 2014 (excluding 2004, when the core general population sample did not have a nurse visit). HSE is an annual household survey which uses multilevel stratification to select a new, nationally-representative sample each year. These years were selected because identical variables have been coded consistently from 2003 onwards; 2014 was the most recent dataset available in the UK Data Archive at the time the analyses commenced. Data were collected first by an interviewer, who also measured height and weight; a nurse visit followed for participants who agreed. The nurse asked further questions and took biophysical measurements, including blood pressure. The survey varied in size each year from around 9,000 to 16,000 households in the study sample.[26] Research ethics approval was obtained prior to each survey from the relevant research ethics committees. Participants gave informed consent at each stage of the survey.

The study sample comprised adults who had a nurse visit; the analytical sample was adults who had observations for indoor temperature and self-rated health (Supplementary Figure S1). The analytic sample (n=74,736) was compared with those excluded from the study sample (n=2,065, Supplementary Table S1). There were no statistically significant differences between the analytic sample and those excluded regarding mean monthly outdoor temperatures by region (data not shown). However, among the categorical variables, only respiratory disease had non-significant differences in their distribution within the two samples. The analytic sample comprised 55% women, while 74% of the excluded participants were women ($P<0{\cdot}01$). The excluded survey participants had a younger age profile than the analytic sample; were less likely to be in the top two income quintiles; were more likely to live in social housing; but were more likely to have degree level qualifications. The analytic sample were more likely to own their homes, and to have a longstanding illness or cardiovascular condition.

Data

General health was a self-assessed variable, referred to as self-rated health. It was recorded at the interview stage, prior to the nurse visit. The participants selected one of the five answer options *“Very good, good, fair, bad or very bad?”* to the question: *“How is your health in general?”* For this analysis, general health was dichotomised into ‘good’, which

For peer review only - http://bmjopen.bmj.com/site/about/guidelines.xhtml

encompassed the categories very good and good, and ‘poor’, which included fair, bad and very bad. Fair was placed in the ‘poor’ category rather than the ‘good’ category as the distribution of general health is highly skewed: 74% of participants reported good or very good general health.

The other self-reported health data used in these analyses included the presence of: limiting or non-limiting longstanding illness; a respiratory condition; or a cardiovascular condition. Other data collected at the interview included gender; age; socio-economic data (National Statistics-Socio-Economic Classification of occupation in five categories (NS-SEC5); education level, categorised as degree or equivalent, other qualification, or no qualification; and quintile of equivalised household income); and housing information (number of occupants and housing tenure).

For the variables NS-SEC5, income and tenure, answers in the ‘other’ category, or categories outside of the main analytical categories were grouped into an additional category of ‘other or missing’ and included in the models. This was because there were many participants in those categories and excluding them would have substantially decreased the sample size.

Indoor air temperature was measured by the nurse, using a digital thermometer with a probe, prior to measuring blood pressure. The thermometer was placed on a surface near the blood pressure equipment, away from a radiator and out of direct sunlight. Nurses were recommended that the probe hang over the edge of the table.

Mean monthly temperature data by region were obtained from the Met Office website. The data were publicly available. The Met Office is a governmental agency in the UK which provides weather forecasts and warnings. The data were measured according to their standard protocol at weather stations 40 kilometres apart across the country, every hour.

Data sharing

The pseudonymised HSE data from each survey are deposited in the UK Data Archive and are available to be downloaded from the UK Data Service at: beta.ukdataservice.ac.uk/datacatalogue/series/series?id=2000021.

Statistical analysis

The analysis was run in Stata v15 (StataCorp, 2017). Models were built to test each hypothesis. Each model used the same analytical sample. The models were built using maximum likelihood estimation, and the best models were selected using the likelihood ratio test. Age was grouped for analysis as 16-24 then 10y groups to 75+, to avoid prior assumptions of the shape of the relationship.

To test the association of indoor temperature with general health, logistic regression models were created with indoor temperature as the explanatory variable, and general self-reported health as the outcome variable. The model was stratified by indoor temperature to assess if the direction of the association differed across the range of indoor temperatures.

To test for associations between socioeconomic and housing variables and linear indoor temperature, linear regression models were created with socioeconomic and housing variables as explanatory variables. Missing data among the covariates were included in the regression as an additional category; results are not shown in the tables.

For peer review only - http://bmjopen.bmj.com/site/about/guidelines.xhtml

The unadjusted coefficients of the variables are reported, as well as the coefficients after adjusting for other socioeconomic and housing variables. Collinearity was assessed by looking for reversal of the sign of the coefficients, and the variance inflation factor.

Results

The analytical sample consisted of 74,736 participants, 97·3% of the study sample. Their characteristics are shown in Supplementary Table S1. The indoor temperature ranged from 7·5°C to 36·8°C, with a mean of 20·7°C (SD 2·3). For both indoor and outdoor temperature, most of the variation was attributable to month. 10% of households had temperatures below the UK government's recommended minimum of 18°C.

<Insert Table 1 around here>

Socioeconomic and housing variables and indoor temperature

Households with more occupants and higher equivalised household incomes were associated with a lower odds of an indoor temperature <18°C (Table 1) and higher mean indoor temperatures (Tables 2 and S1). Higher educational attainment, and private sector rented houses were associated with lower mean indoor temperatures (Table 2) and a higher odds of the indoor temperature being <18°C (Table 1). NS-SEC5 was variably associated with low temperature (reduced odds among intermediate but higher odds among small employers) and with mean indoor temperature (positive association in lower supervisory and technical households but inverse association among small employers) in the fully adjusted models, compared with those in managerial and professional occupations.

<Insert Table 2 around here>

For peer review only

For peer review only - http://bmjopen.bmj.com/site/about/guidelines.xhtml

Table 1 - Logistic regression models of associations of socioeconomic and housing variables with low indoor temperature (<18°C)

	Minimally adjusted[a]				Fully adjusted[b]			
	OR	95% CI		P	OR	95% CI		P
**Household size**								
1	1				1			
2	0·58	0·54	0·63	<0·001	0·59	0·54	0·63	<0·001
3	0·46	0·41	0·51	<0·001	0·46	0·41	0·51	<0·001
4	0·38	0·34	0·43	<0·001	0·38	0·34	0·43	<0·001
5+	0·37	0·32	0·44	<0·001	0·36	0·31	0·43	<0·001
**Tenure**								
Owner-occupied	1				1			
Privately rented	1·49	1·33	1·68	<0·001	1·27	1·12	1·43	<0·001
Socially rented	1·10	1·00	1·20	0·056	0·94	0·85	1·05	0·264
**NS-SEC5**								
Managerial & professional	1				1			
Intermediate	0·90	0·83	0·98	0·015	0·92	0·84	1·00	0·047
Small employers	1·17	1·06	1·29	0·003	1·20	1·09	1·33	<0·001
Lower supervisory & technical	0·91	0·81	1·01	0·083	0·93	0·83	1·05	0·236
Semi-routine	1·00	0·94	1·08	0·901	1·02	0·94	1·11	0·585
**Education**								
Degree	1				1			
Qualification below degree	0·91	0·85	0·98	0·017	0·92	0·85	1·00	0·045
No qualification	0·90	0·82	0·99	0·029	0·84	0·75	0·93	0·001
**Equivalised household income quintile**								
Lowest quintile	1				1			
2nd quintile	0·87	0·78	0·98	0·024	0·91	0·81	1·03	0·126
3rd quintile	0·86	0·77	0·96	0·010	0·88	0·78	0·99	0·030
4th quintile	0·84	0·75	0·95	0·004	0·83	0·73	0·94	0·003
Highest quintile	0·86	0·77	0·97	0·013	0·77	0·67	0·88	<0·001

[a] Minimally adjusted models: adjusted for outdoor temperature and age group.

[b] Fully adjusted models: mutually adjusted for all socioeconomic and housing variables in this table.

For peer review only

For peer review only - http://bmjopen.bmj.com/site/about/guidelines.xhtml

Table 2 - Linear regression models with socioeconomic and housing variables as explanatory variables for indoor temperature

	Minimally adjusted[a]				Fully adjusted[b]			
	β	95% CI		P	β	95% CI		P
**Household size**								
1	Ref							
2	0·41	0·35	0·46	<0·001	0·43	0·38	0·49	<0·001
3	0·63	0·56	0·71	<0·001	0·65	0·58	0·73	<0·001
4	0·74	0·66	0·82	<0·001	0·76	0·68	0·85	<0·001
5+	0·79	0·68	0·90	<0·001	0·81	0·70	0·93	<0·001
**Tenure**								
Owner-occupied	Ref							
Privately rented	-0·23	-0·32	-0·14	<0·001	-0·11	-0·20	-0·02	0·017
Socially rented	0·13	0·06	0·19	<0·001	0·21	0·14	0·28	<0·001
**NS-SEC5**								
Managerial and professional occupations	Ref							
Intermediate	0·08	0·03	0·13	0·003	0·03	-0·03	0·08	0·298
Small employers	-0·05	-0·12	0·02	0·177	-0·11	-0·18	-0·04	0·001
Lower supervisory and technical	0·18	0·11	0·24	<0·001	0·09	0·02	0·16	0·010
Semi-routine	0·06	0·01	0·11	0·020	-0·04	-0·09	0·01	0·128
**Education**								
Degree	Ref							
Qualification below degree	0·16	0·11	0·21	<0·001	0·14	0·08	0·19	<0·001
No qualification	0·22	0·16	0·29	<0·001	0·22	0·15	0·29	<0·001
**Income quintile**								
Lowest quintile	Ref							
2nd quintile	0·03	-0·05	0·11	0·468	0·04	-0·05	0·12	0·402
3rd quintile	0·02	-0·06	0·10	0·631	0·07	-0·01	0·16	0·081
4th quintile	0·01	-0·07	0·09	0·843	0·11	0·02	0·19	0·016
Highest quintile	-0·04	-0·12	0·05	0·402	**0·15**	**0·06**	**0·24**	**0·001**

[a] Minimally adjusted models: adjusted for outdoor temperature and age group.
[b] Fully adjusted models: mutually adjusted for all socioeconomic and housing variables in this table.

For peer review only - http://bmjopen.bmj.com/site/about/guidelines.xhtml

Indoor temperature and health

Each one degree increase in indoor temperature was associated with a 1.4% (95% CI 0.7-2.6) increase in odds of poor health, after adjusting for age and gender (see Fig 1). After adjusting for socioeconomic and housing factors, each degree increase in temperature is associated with a 1.7% (95% CI 0.7-2.6) increase in odds of poor health.

<Insert Figure 1 around here>

A series of regression models were run to examine the impact on the odds of reporting poor health associated with indoor temperature of demographic, housing, and socio-economic factors (Table 3). Adjusting for household size, household size and tenure, or for equivalised household income increased the odds ratio by 43%, 7% or 12% respectively. Adjusting for tenure, NS-SEC5, or all three socio-economic factors reduced the odds ratio by 17%, 7% or 11%. The linear association between indoor temperature and reporting poor health remained significant in each model; the final model, including all these covariates, increased the odds ratio by 19%.

<Insert Table 3 around here>

For peer review only

Table 3 - Logistic regression models showing odds ratios of poor health for indoor temperature

Indoor temperature[a,b]	OR	95% CI		P	% change in OR
Model 1: adjusted for age and gender	1·01	1·00	1·02	0·003	Ref
Model 2: Model 1 + Household size	1·02	1·01	1·03	<0·001	+43
Model 3: Model 1 + Tenure	1·01	1·00	1·02	0·014	-17
Model 4: Model 1 + NS-SEC5	1·01	1·00	1·02	<0·005	-7
Model 5: Model 1 + Income	1·02	1·01	1·03	<0·001	+12
Model 6: Model 1 + Education	1·01	1·00	1·02	0·034	-30
Model 7: Model 1 + housing variables	1·02	1·01	1·02	0·002	+7
Model 8, Model 1 + socioeconomic variables	1·01	1·00	1·02	0·007	-11
Model 9: Model 1 + housing and socioeconomic variables	1·02	1·01	1·03	<0·001	+19

[a] Indoor temperature was linear in these models.
[b] Models are listed in order of increasing likelihood. All models adjusted for age group and gender; Model 7 as Model 1 plus household size and tenure; Model 8 as Model 1 plus NS-SEC5, equivalised income quintiles, and education; Model 9 included all the variables.
[c] The % change in OR compares each models with model 1.

Discussion

The results showed an association between higher indoor temperatures and increased odds of poor self-rated health. This has also been shown recently in a small number of individuals in Cornwall, UK: participants with poorer health maintained their homes at higher temperatures.[27] However, the relationship between socioeconomic and housing factors with indoor temperature was complicated, with different measures having different directions of association with indoor temperature. A small study in Japan, using perceived thermal comfort not measured temperature, found that people who reported living in cold homes had a higher risk of frailty - but only among those who were dissatisfied with their economic situation.[28]

It is not clear from this research why temperatures are higher among households with worse self-rated health and whether this is due to personal preferences and behaviours; advice from medical or other professionals to maintain a warmer home; the difference in the type of building they occupy (i.e. dwellings that are typically warmer such as smaller houses or flats, and social housing rather than privately rented); or some combination of these. Figure 2 shows a conceptual framework for the way in which these variables affect the associations.

<Figure 2 around here>

The strengths of this study are the large number of participants, who were nationally-representative of the non-institutionalised population living in England. The temperature measurements were taken using a standard protocol across the time period but were not the focus of the surveys, so the likelihood of differential response rates systematically

For peer review only - http://bmjopen.bmj.com/site/about/guidelines.xhtml

associated with indoor temperature is very small. Unlike the study by Armstrong and colleagues, we report data based on individuals, rather than ecological analyses.[29]

Limitations include the significant differences in many characteristics between the analytic sample, who had indoor measurements, and those excluded because there were no measurements. The latter comprises those who were ineligible for blood pressure measurement (for example being pregnant) or declined the measurement. However, fewer than 3% of the study sample were excluded; the analytic sample is therefore almost identical to the study sample overall. It should also be noted that there was a single time point indoor temperature measurement for each participant wherever they were 'hosting' the survey nurse, generally in the living room or kitchen. Huebner and colleagues have shown four different profiles in living room temperature variability across the day, varying from bimodal (morning and evening peaks), steadily increasing (shallow and steep) to flat with little variation.[30]

While the subjectivity of self-rated health makes it vulnerable to information bias, it has been shown to be highly predictive of future mortality and health outcomes, and is used frequently across the scientific literature.[31] Given this is the first study of its kind, and there is less evidence pointing at more specific health outcomes, the broadness of general health as the outcome was perhaps a strength rather than a weakness. The differences between the entire sample and those with complete data on self-rated health and temperature was primarily due to non-response to the nurse visit among interviewees. This was dealt with by use of non-response weighting from HSE 2003 onwards; response rates prior to that were high, though falling.[32]

While guidelines on healthy indoor temperatures have been issued by the World Health Organisation (WHO) and the UK Public Health England, they appear arbitrary and the WHO itself describe its own temperature recommendations as *'[scientifically] weak and not well-known to the public or policy makers'*.[33-35] Since 2014, the recommendation from the UK government is for a minimum of 18°C in all rooms, a change from the previous 21°C for living rooms.[29] The government attributed the change to a review of evidence, awareness of climate change and financial difficulties, and acceptability (*"We know that people have strong feelings about their homes and don't want to be told what to do in them")*.[35] Further research is needed to understand the temporality and mechanism of the association.

A recent study in England found no impact of Winter Fuel Payment eligibility on indoor temperature [36], but no study has examined at individual level the impacts of energy efficiency retrofits on indoor temperature, residents' heating-relate behaviours, or health. When linked data are available, we intend to examine the associations of indoor temperature with subsequent hospital admissions and mortality. This will be important for assessing and making health-related policy recommendations for indoor temperature. Future research in this area could take advantage of internet-enabled 'smart' thermostats.

For peer review only - http://bmjopen.bmj.com/site/about/guidelines.xhtml

Figure captions

Figure 1 - Probability of poor health for each degree increase in indoor temperature

Figure 2 - Conceptual framework for temperature-health association

For peer review only

For peer review only - http://bmjopen.bmj.com/site/about/guidelines.xhtml

Acknowledgements

We thank the nurses and participants in the Health Surveys for England, and our colleagues at NatCen Social Research and UCL.

Author contributions

This work was conducted as part of JS-K's MSc in Social Epidemiology. She led on conducting the literature search, designing and conducting the analyses, creating the figures, and interpreting the data. AM and JM contributed advice to the dissertation. JS-K and JM wrote the initial draft of the manuscript. IH updated the literature search and contributed to analysis and preparation of the manuscript. All authors contributed to subsequent drafts, approved the final manuscript, and are accountable for the accuracy and integrity of all aspects of the work.

Patient and Public Involvement

This study does not involve patients as the data come from a cross-section of the general population, regardless of their involvement (or not) with any health services. The public were not involved in the design of this study but the Health Survey for England itself has an Advisory Group that includes stakeholders from local government, public health, the Department of Health and Social Care, Public Health England, NHS Digital, and academe.

Age UK, Kilburn Older Voices (KOVE), the Department of Business, Energy and Industrial Strategy (BEIS), the Met Office, NatCen Social Research, and Public Health England will help with the dissemination of these findings.

Role of the funding source

This work was supported by NHS Digital, which funds the HSE, also fund AM & JM to work on the annual HSE. This work was also supported by the EPSRC [EP/R035288/1] to fund IH to work on home energy efficiency. NHS Digital and EPSRC played no part in the planning, conduct, or interpretation of these analyses; the drafting of the manuscript; nor the decision to publish.

Conflict of interest statement

Professor Mindell and Dr. Moody report grants from NHS Digital during the conduct of the study; Dr. Sutton-Klein has nothing to disclose. Dr. Hamilton report grants from the Engineering and Physical Sciences Research Council (EPSRC).

For peer review only - http://bmjopen.bmj.com/site/about/guidelines.xhtml

For peer review only

References

1 Analitis A, Katsouyanni K, Biggeri A*, et al.* Effects of Cold Weather on Mortality: Results From 15 European Cities Within the PHEWE Project. *American Journal of Epidemiology* 2008;**168**:1397-408.
2 Tham S, Thompson R, Landeg O*, et al.* Indoor temperature and health: a global systematic review. *Public Health* 2020;**179**:9-17.
3 The Health Impacts of Cold Homes and Fuel Poverty. *Friends of the Earth*: Marmot Review Team 2011.
4 Hammel HT, Pierce J. Regulation of internal body temperature. *Annual review of physiology* 1968;**30**:641-710.
5 Mallet ML. Pathophysiology of accidental hypothermia. *QJM : monthly journal of the Association of Physicians* 2002;**95**:775-85.
6 ONS. Excess Winter Mortality in England and Wales : 2018/19 (Provisional) and 2017/18 (Final). ONS, 2019.
7 Liddell C, Morris C, Thomson H*, et al.* Excess winter deaths in 30 European countries 1980–2013: a critical review of methods. . *J Public Health* 2015;**38**:806-14.
8 Laake K, Sverre.J.M. Winter Excess Mortality: A Comparison between Norway and England. *Age and Ageing* 1996;**25**:343-8.
9 Fowler T, Southgate RJ, Waite T*, et al.* Excess winter deaths in Europe: a multi-country descriptive analysis. *Eur J Public Health* 2015;**25**:339-45.
10 Healy JD. Excess winter mortality in Europe: a cross country analysis identifying key risk factors. *Journal of Epidemiology and Community Health* 2003;**57**:784-9.
11 Collins KJ. Low indoor temperatures and morbidity in the elderly. *Age Ageing* 1986;**15**:212-20.
12 Clinch JP, Healy JD. Housing standards and excess winter mortality. *Journal of Epidemiology and Community Health* 2000;**54**:719-20.
13 Howden-Chapman P, Matheson A, Crane J*, et al.* Effect of insulating existing houses on health inequality: cluster randomised study in the community. *Bmj* 2007;**334**:460.
14 Lloyd EL, McCormack C, McKeever M*, et al.* The effect of improving the thermal quality of cold housing on blood pressure and general health: a research note. *J Epidemiol Community Health* 2008;**62**:793-7.
15 Ormandy D, Ezratty V. Health and thermal comfort: From WHO guidance to housing strategies. . *Energy Policy* 2012;**49**:116-21.
16 Evans J, Hyndman S, Stewart-Brown S*, et al.* An epidemiological study of the relative importance of damp housing in relation to adult health. *Journal of Epidemiology and Community Health* 2000;**54**:677-86.
17 Wilkinson P, Landon M, Armstrong B*, et al.* Cold comfort: the social and environmental determinants of excess winter death in England, 1986-1996. York, UK 2001.
18 Hamilton IG, O'Sullivan A, Huebner G*, et al.* Old and cold? Findings on the determinants of indoor temperatures in English dwellings during cold conditions. *Energy and Buildings* 2017;**141**:142-57.
19 Jevons R, Carmichael C, Crossley A*, et al.* Minimum indoor temperature threshold recommendations for English homes in winter – A systematic review. *Public Health* 2016;**136**:4-12.

For peer review only - http://bmjopen.bmj.com/site/about/guidelines.xhtml

20 Huebner GM, Hamilton I, Chalabi Z*, et al.* Comparison of indoor temperatures of homes with recommended temperatures and effects of disability and age: an observational, cross-sectional study. *BMJ Open* 2018;**8**:e021085.
21 Lelkes O, Zolyomi E. Quality of housing and the link to income. Research note n 2. Brussel: European Commission 2009.
22 Tunstall R, Bevan M, Bradshaw J*, et al.* The links between housing and poverty: an evidence review. Joseph Rowntree Foundation 2013.
23 Marshall C, Moody A, Hamilton I*, et al.* Changes in heating of homes from 1991 to 2011 using data from the ONS-Longitudinal Study. *European Network for Housing Research Conference* 2016.
24 Ministry of Housing CLG. English Housing Survey 2016 to 2017: headline report. London 2018.
25 Oreszczyn T, Hong SH, Ridley I*, et al.* Determinants of winter indoor temperatures in low income households in England. *Energy and Buildings* 2006;**38**:245-52.
26 Mindell J, Biddulph JP, Hirani V*, et al.* Cohort Profile: The Health Survey for England. *International Journal of Epidemiology* 2012;**41**:1585-93.
27 Pollard A, Jones T, Sherratt S*, et al.* Use of Simple Telemetry to Reduce the Health Impacts of Fuel Poverty and Living in Cold Homes. *International Journal of Environmental Research and Public Health* 2019;**16**:2853.
28 Nakajima Y, Schmidt SM, Malmgren Fänge A*, et al.* Relationship between Perceived Indoor Temperature and Self-Reported Risk for Frailty among Community-Dwelling Older People. *International Journal of Environmental Research and Public Health* 2019;**16**:613.
29 Armstrong B, Bonnington O, Chalabi Z*, et al.* The impact of home energy efficiency interventions and winter fuel payments on winter- and cold-related mortality and morbidity in England: a natural equipment mixed-methods study. *Southampton (UK): NIHR Journals Library; 2018 Oct. Public Health Research, No. 6.11.* www.ncbi.nlm.nih.gov/books/NBK532137/ (accessed 3 Dec 2019).
30 Huebner GM, McMichael M, Shipworth D*, et al.* The shape of warmth: temperature profiles in living rooms. *Building Research & Information* 2015;**43**:185-96.
31 Simon JG, De Boer JB, Joung IM*, et al.* How is your health in general? A qualitative study on self-assessed health. *Eur J Public Health* 2005;**15**:200-8.
32 Mindell JS, Giampaoli S, Goesswald A*, et al.* Sample selection, recruitment and participation rates in health examination surveys in Europe – experience from seven national surveys. *BMC Medical Research Methodology* 2015;**15**:78.
33 Housing, energy and thermal comfort: a review of 10 countries within the WHO European Region. World Health Organization 2007.
34 The Effects of the Indoor Housing Climate on the Health of the Elderly: Report on [ie, Of] a WHO Working Group [held At] Graz, Austria, 20-24 September 1982. WHO Regional Office for Europe 1984.
35 Bone DA, Carmichael K, Wookey R. The Cold Weather Plan for England: Protecting health and reducing harm from cold weather. London: Public Health England 2014.
36 Angelini V, Daly M, Moro M*, et al.* The effect of the Winter Fuel Payment on household temperature and health: a regression discontinuity design study. *Southampton (UK): NIHR Journals Library; 2019 Jan. Public Health Research, No 7.1*. https://www.ncbi.nlm.nih.gov/books/NBK535694/

For peer review only - http://bmjopen.bmj.com/site/about/guidelines.xhtml

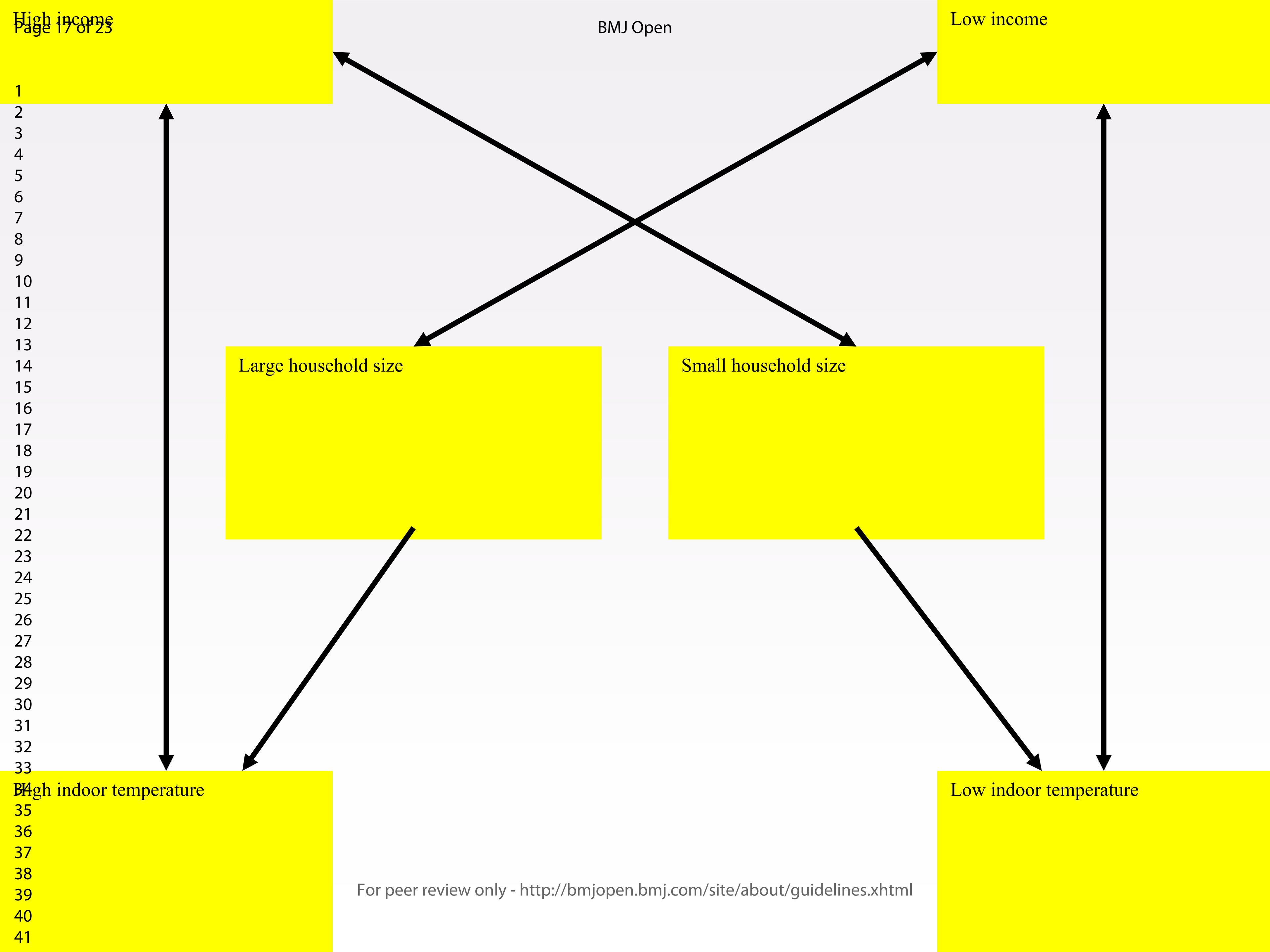

For peer review only - http://bmjopen.bmj.com/site/about/guidelines.xhtml

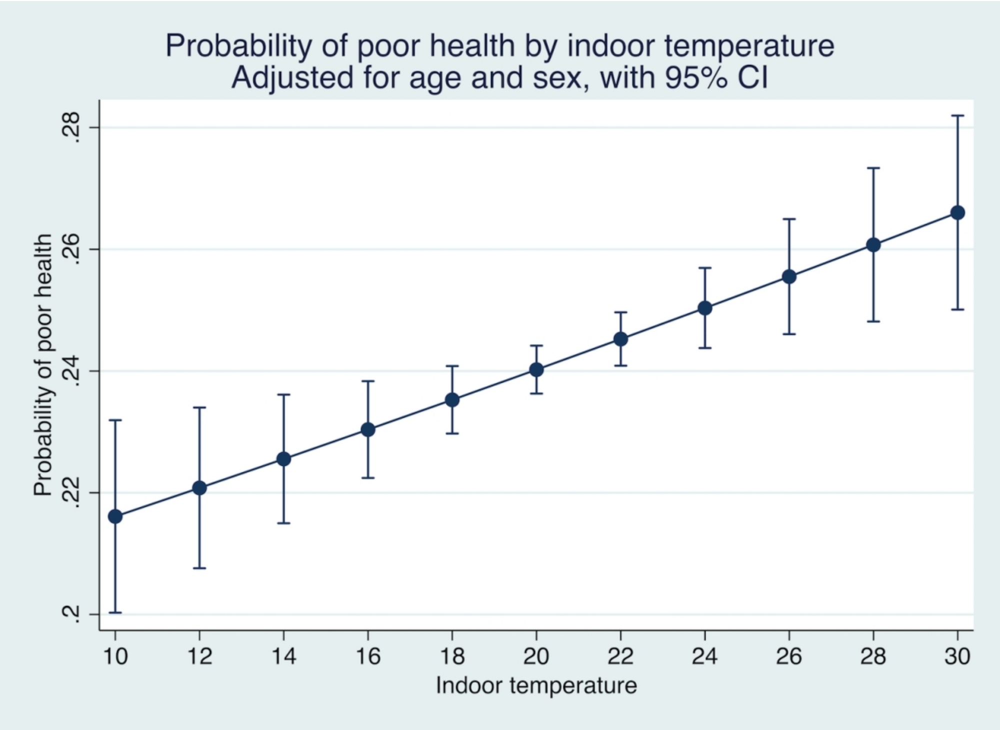

Probability of poor health for each degree increase in indoor temperature

123x90mm (300 x 300 DPI)

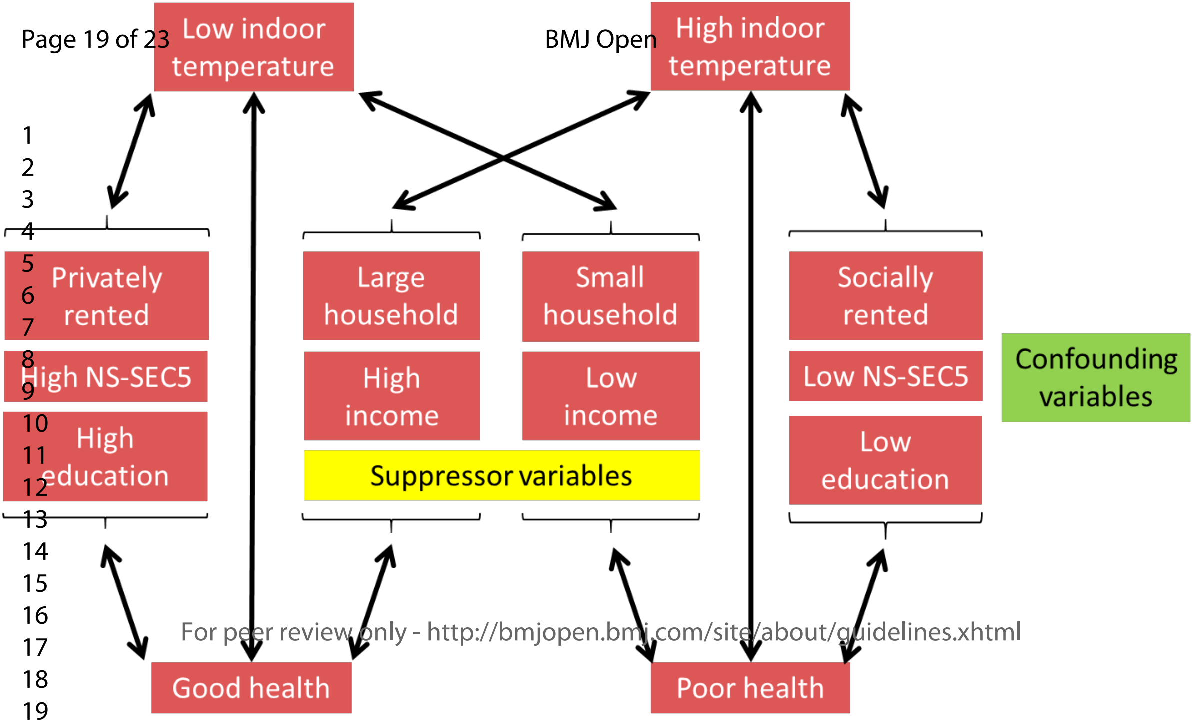

For peer review only - http://bmjopen.bmj.com/site/about/guidelines.xhtml

Figure S1. Flow diagram showing selection of data into analytical sample

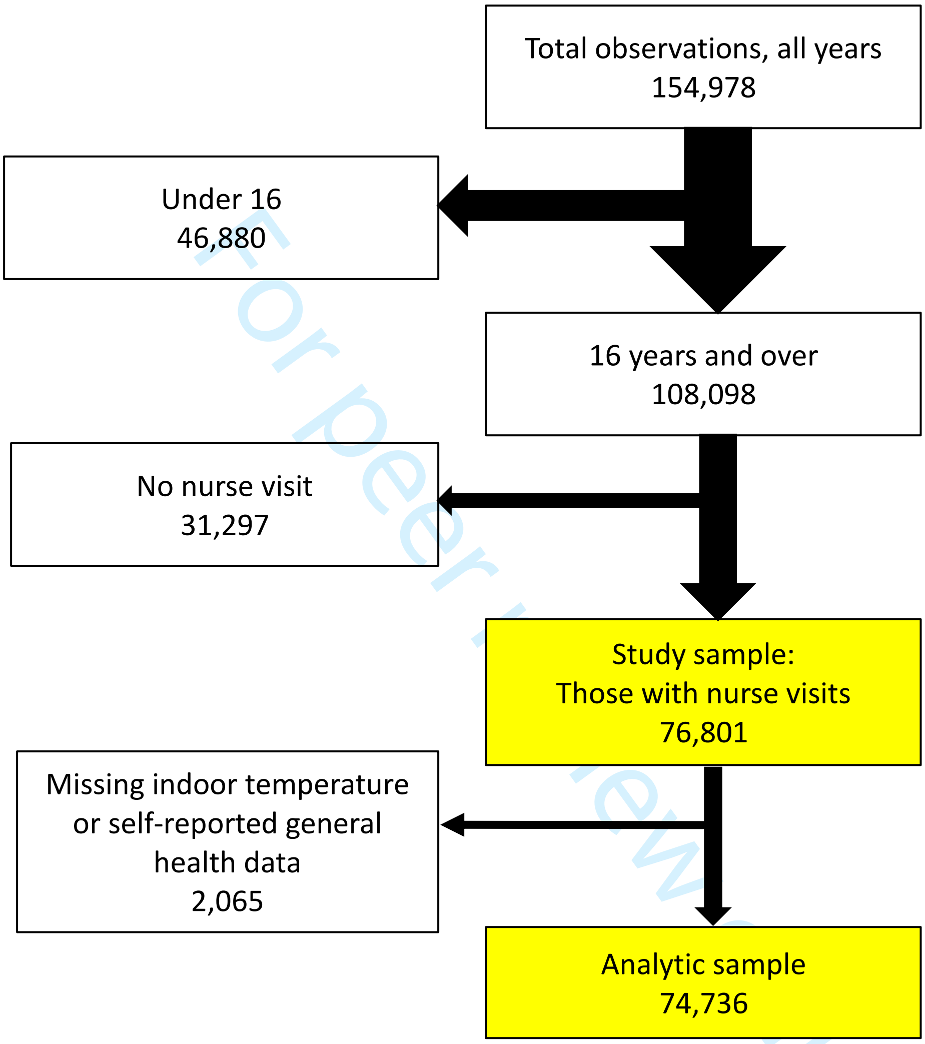

PPI statement – HEAT

This study does not involve patients as the data come from a cross-section of the general population, regardless of their involvement (or not) with any health services. The public were not involved in the design of this study but the Health Survey for England itself has an Advisory Group that includes stakeholders from local government, public health, the Department of Health and Social Care, Public Health England, NHS Digital, and academe.

Age UK, Kilburn Older Voices (KOVE), the Department of Business, Energy and Industrial Strategy (BEIS), the Met Office, NatCen Social Research, and Public Health England will help with the dissemination of these findings.

For peer review only

STROBE Statement—Checklist of items that should be included in reports of ***cross-sectional studies***

	Item No	Recommendation	This manuscript
**Title and abstract**	1	(*a*) Indicate the study's design with a commonly used term in the title or the abstract	The word 'survey' in the title; 'cross-sectional surveys' in the abstract
		(*b*) Provide in the abstract an informative and balanced summary of what was done and what was found	Yes
**Introduction**			
Background/rationale	2	Explain the scientific background and rationale for the investigation being reported	Yes
Objectives	3	State specific objectives, including any prespecified hypotheses	Yes
**Methods**			
Study design	4	Present key elements of study design early in the paper	Yes
Setting	5	Describe the setting, locations, and relevant dates, including periods of recruitment, exposure, follow-up, and data collection	Yes
Participants	6	(*a*) Give the eligibility criteria, and the sources and methods of selection of participants	Yes
Variables	7	Clearly define all outcomes, exposures, predictors, potential confounders, and effect modifiers. Give diagnostic criteria, if applicable	Yes
Data sources/ measurement	8*	For each variable of interest, give sources of data and details of methods of assessment (measurement). Describe comparability of assessment methods if there is more than one group	Yes
Bias	9	Describe any efforts to address potential sources of bias	Yes
Study size	10	Explain how the study size was arrived at	Yes
Quantitative variables	11	Explain how quantitative variables were handled in the analyses. If applicable, describe which groupings were chosen and why	Yes
Statistical methods	12	(*a*) Describe all statistical methods, including those used to control for confounding	Yes
		(*b*) Describe any methods used to examine subgroups and interactions	Yes

For peer review only - http://bmjopen.bmj.com/site/about/guidelines.xhtml

		(*c*) Explain how missing data were addressed	Yes
		(*d*) If applicable, describe analytical methods taking account of sampling strategy	Yes
		(*e*) Describe any sensitivity analyses	No additional sensitivity analysis undertaken
**Results**			
Participants	13*	(a) Report numbers of individuals at each stage of study—eg numbers potentially eligible, examined for eligibility, confirmed eligible, included in the study, completing follow-up, and analysed	Yes
		(b) Give reasons for non-participation at each stage	Yes
		(c) Consider use of a flow diagram	Provided as supplementary material Figure S1
Descriptive data	14*	(a) Give characteristics of study participants (eg demographic, clinical, social) and information on exposures and potential confounders	Table 1
		(b) Indicate number of participants with missing data for each variable of interest	N/A
Outcome data	15*	Report numbers of outcome events or summary measures	N/A
Main results	16	(*a*) Give unadjusted estimates and, if applicable, confounder-adjusted estimates and their precision (eg, 95% confidence interval). Make clear which confounders were adjusted for and why they were included	95% CIs provided for minimally-adjusted (age and outdoor temperature and fully adjusted models
		(*b*) Report category boundaries when continuous variables were categorized	Yes
		(*c*) If relevant, consider translating estimates of relative risk into absolute risk for a meaningful time period	N/A
Other analyses	17	Report other analyses done—eg analyses of subgroups and interactions, and sensitivity analyses	Yes
**Discussion**			
Key results	18	Summarise key results with reference to study objectives	Yes
Limitations	19	Discuss limitations of the study, taking into account sources of potential bias or imprecision. Discuss both direction and magnitude of any potential bias	Yes

For peer review only

For peer review only - http://bmjopen.bmj.com/site/about/guidelines.xhtml

Interpretation	20	Give a cautious overall interpretation of results considering objectives, limitations, multiplicity of analyses, results from similar studies, and other relevant evidence	Yes
Generalisability	21	Discuss the generalisability (external validity) of the study results	Yes
**Other information**			
Funding	22	Give the source of funding and the role of the funders for the present study and, if applicable, for the original study on which the present article is based	Yes

*Give information separately for exposed and unexposed groups.

Note: An Explanation and Elaboration article discusses each checklist item and gives methodological background and published examples of transparent reporting. The STROBE checklist is best used in conjunction with this article (freely available on the Web sites of PLoS Medicine at http://www.plosmedicine.org/, Annals of Internal Medicine at http://www.annals.org/, and Epidemiology at http://www.epidem.com/). Information on the STROBE Initiative is available at www.strobe-statement.org.

For peer review only

BMJ Open

Associations between indoor temperature, self-rated health and socio-economic position in a cross-sectional study of adults in England

Journal:	*BMJ Open*
Manuscript ID	bmjopen-2020-038500.R1
Article Type:	Original research
Date Submitted by the Author:	12-Aug-2020
Complete List of Authors:	Sutton-Klein, Joanna; UCL, Epidemiology and public health<br>Moody, Alison; UCL, Epidemiology and public health<br>Hamilton, Ian; UCL, UCL Energy Institute<br>Mindell, Jennifer; UCL, Epidemiology & Public Health
**Primary Subject Heading**:	Public health
Secondary Subject Heading:	Epidemiology, Health policy
Keywords:	EPIDEMIOLOGY, PUBLIC HEALTH, SOCIAL MEDICINE

SCHOLARONE™
Manuscripts

I, the Submitting Author has the right to grant and does grant on behalf of all authors of the Work (as defined in the below author licence), an exclusive licence and/or a non-exclusive licence for contributions from authors who are: i) UK Crown employees; ii) where BMJ has agreed a CC-BY licence shall apply, and/or iii) in accordance with the terms applicable for US Federal Government officers or employees acting as part of their official duties; on a worldwide, perpetual, irrevocable, royalty-free basis to BMJ Publishing Group Ltd ("BMJ") its licensees and where the relevant Journal is co-owned by BMJ to the co-owners of the Journal, to publish the Work in this journal and any other BMJ products and to exploit all rights, as set out in our licence.

The Submitting Author accepts and understands that any supply made under these terms is made by BMJ to the Submitting Author unless you are acting as an employee on behalf of your employer or a postgraduate student of an affiliated institution which is paying any applicable article publishing charge ("APC") for Open Access articles. Where the Submitting Author wishes to make the Work available on an Open Access basis (and intends to pay the relevant APC), the terms of reuse of such Open Access shall be governed by a Creative Commons licence – details of these licences and which Creative Commons licence will apply to this Work are set out in our licence referred to above.

Other than as permitted in any relevant BMJ Author's Self Archiving Policies, I confirm this Work has not been accepted for publication elsewhere, is not being considered for publication elsewhere and does not duplicate material already published. I confirm all authors consent to publication of this Work and authorise the granting of this licence.

For peer review only

Associations between indoor temperature, self-rated health and socio-economic position in a cross-sectional study of adults in England

Authors:

Dr Joanna Sutton-Klein, MB ChB, MSc, Health and Social Surveys Research Group, Research Dept of Epidemiology and Public Health, UCL, 1-19 Torrington Place, London WC1E 6BT, UK. ORCID 0000-0002-7784-0986

Alison Moody, MSc, Health and Social Surveys Research Group, Research Dept of Epidemiology and Public Health, UCL, 1-19 Torrington Place, London WC1E 6BT, UK. ORCID 0000-0002-8199-4830

Dr Ian Hamilton, PhD, UCL Energy Institute, Bartlett Faculty of the Built Environment, UCL, 14 Upper Woburn Place, London, WC1H 0NN, UK. ORCID 0000-0003-2582-2361

Prof Jennifer S. Mindell, PhD, Health and Social Surveys Research Group, Research Dept of Epidemiology and Public Health, UCL, 1-19 Torrington Place, London WC1E 6BT, UK. ORCID 0000-0002-7604-6131

Corresponding author:

Dr Ian Hamilton, UCL Energy Institute, Bartlett Faculty of the Built Environment, UCL, Central House, 14 Upper Woburn Place, London, WC1H 0NN

Email: i.hamilton@ucl.ac.uk

Telephone: +44-20-3108-5982

Research report manuscript for *BMJ Open*

Word count: 3,576

Tables: 3 plus one supplementary table

Figures: 2 plus one supplementary figure

References: 58

Subject areas: Public health; Health policy

Journal keywords: Health status, Socio-economic inequalities, Temperature

Funding

AM & JM are funded by NHS Digital to work on the annual HSE. IH is funded by EPSRC [EP/R035288/1] to work on building energy efficiency. NHS Digital and EPSRC played no part in the planning, conduct, or interpretation of these analyses; the drafting of the manuscript; nor the decision to publish.

For peer review only - http://bmjopen.bmj.com/site/about/guidelines.xhtml

Abstract

Objective

Excess winter deaths (EWDs) are a major public health concern in England and Wales, with an average of 20,000 deaths per year since 2010. Feeling cold at home during winter is associated with reporting poor general health; cold and damp homes have greater prevalence in lower socioeconomic groups. Overheating in the summer also has adverse health consequences. This study evaluates the association between indoor temperature and general health and the extent to which this is affected by socioeconomic and household factors.

Design

Cross-sectional study.

Setting

England.

Participants

Secondary data of 74,736 individuals living in England that took part in the Health Survey for England (HSE) between 2003 and 2014. The HSE is an annual household survey which uses multilevel stratification to select a new, nationally-representative sample each year. The study sample comprised adults who had a nurse visit; the analytical sample was adults who had observations for indoor temperature and self-rated health.

Results

Using both logistic and linear regression models to examine indoor temperature and health status, adjusting for socio-economic and housing factors, the study found an association between poor health and higher indoor temperatures. Each one degree increase in indoor temperature was associated with a 1·7% (95% Confidence Interval (CI) 0·7%-2·6%) increase in the odds of poor health. After adjusting for income, education, employment type, household size and home ownership, the odds ratio (OR) of poor health for each degree temperature rise increased by 19%, to 1·02 (95%CI 1·01-1·03 P<0·01).

Conclusion

People with worse self-reported health had higher indoor temperatures after adjusting for household factors. People with worse health may have chosen to maintain warmer environments or been advised to. However, other latent factors, such as housing type and energy performance could have an effect.

Article summary: Strengths and limitations of this study

- This study is based on data from 74,736 individuals, randomly sampled from the general population to be nationally-representative of the population in England.
- Temperature measurements were taken using a standard protocol across the 10 years.
- Analyses used individual-level data, not ecological comparisons.
- Indoor temperatures were measured only once per participant.
- Data are cross-sectional, so causality cannot be inferred.

For peer review only

For peer review only - http://bmjopen.bmj.com/site/about/guidelines.xhtml

Introduction

Short-term exposure to extreme hot or cold temperatures is known to be acutely harmful. For example, Analitis *et al.* compared the short-term effects of cold on mortality in 15 European cities, finding that a 1°C decrease in outdoor temperature was associated with a 1·35% increase in the daily number of total natural deaths.[1] A recent systematic review has shown the adverse health consequences of high indoor temperatures, above 26°C.[2] However, the long-term effects of exposure to different temperatures are not well understood. Much of the evidence on long-term exposure to more minor deviations in ambient temperature is overwhelmingly drawn from ecological studies.[3–5]

There were 49,410 excess winter deaths (EWDs) in England and Wales over the 2017-18 winter.[6] Although EWDs were significantly lower in 2018-19 (23,200, provisional figure),[6] the large inequalities in EWD index between European countries suggest a major public health problem.[7] Paradoxically, while several studies have found that cooler ambient temperatures are associated with higher mortality on a day-by-day analysis, European countries with colder winters were found to have lower excess winter deaths.[8] Studies have suggested that it is the variation in housing quality and the resulting indoor temperature differences which may instead be the drivers of these differences in health outcomes.[9,10]

Few studies have directly addressed the association between indoor temperature and health in individuals, but a number have studied known correlates of indoor temperature such as damp, insulation, or perceived thermal comfort.[11,12] These studies overwhelmingly found that proxies for cool indoor temperature were associated with worse health.

Several studies have compared the health of individuals before and after housing interventions. Upgraded insulation in New Zealand was associated with increased indoor temperature, and lower levels of poor general health (odds ratio, (OR) 0·50, 95% confidence interval (CI) 0·38-0·68).[13,14] Furthermore, a large pan-European study found that perceived coldness at home was strongly associated with poor general health, with an OR of 2.6 (95% CI 2.1-3.1),[15] a result which was replicated in a study of English adults.[16] A limitation of using perceived thermal comfort as a proxy of indoor temperature is that its subjectivity makes it vulnerable to reverse causation.

Analysis of the 1991 survey of English houses found that the strongest predictors of low indoor temperatures were age of property, absence of central heating, cost of heating, small household size (i.e. fewer occupants), and low income.[17] A 2011 survey of English houses found similar importance of predictors of indoor temperature; that older properties were not the coldest; but that low energy performance ratings and low income households had the lowest temperatures.[18]

A systematic review by Public Health England concluded that living in cold temperatures (below 18°C) may increase the risk of adverse health consequences, particularly for premature mortality in vulnerable populations.[19] However, recommendations for minimum indoor temperature are based on limited evidence.[15] Research has shown that few households are keeping the recommended indoor temperatures (i.e. ≥18°C), particularly vulnerable households.[20] Recently, increasing poverty was associated with higher risk of poor housing within the UK.[21,22] Analysis of Census longitudinal study data has shown a rise in the proportion of homes with central heating from 83% in 1991, to 93% in 2001 and 98% in 2011.[23] The English Housing Survey in 2016 found that prevalence of cavity or solid wall insulation, of double glazing, and of central heating had increased in England to

For peer review only - http://bmjopen.bmj.com/site/about/guidelines.xhtml

49%, 83%, and 92% of households respectively.[24] A study on government interventions found that the installation of heating and insulation was effective at increasing indoor temperatures.[25] There are also deaths in England due to excessive temperatures during heatwaves,[26] so both high and low indoor temperatures warrant studying in relation to associations with health, although cold is the primary focus because that is the dominant exposure in England.[27]

The aim of our study was to investigate the relationship between indoor temperature and general health, and to examine the extent to which socioeconomic and housing variables explain any association found. We hypothesised that: socioeconomic and housing factors would be associated with indoor temperature; low indoor temperature would be associated with worse general health; and the association between temperature and general health would be stronger with decreasing socioeconomic status.

Methods

Study design

This study is based on the Health Survey for England (HSE), an annual, cross-sectional, household, health examination survey which has run since 1991. A new, nationally-representative sample of the free-living general population is selected each year, using a stratified, two-stage selection process. The sampling frame is the small addresses Postal Address File. The survey varied in size each year from around 9,000 to 16,000 households in the study sample. Data were collected first by an interviewer, who also measured height and weight; a nurse visit followed for participants who agreed. The nurse asked further questions and took biophysical measurements, including blood pressure. More details are reported elsewhere,[28,29] including the Methods report for the annual report published each year (e.g.[30]).

For this survey, we used data from each year HSE 2003 to HSE 2014 excluding 2004, when the core general population sample did not have a nurse visit. These years were selected because identical variables have been coded consistently from 2003 onwards; 2014 was the most recent dataset available in the UK Data Archive at the time the analyses commenced.

Participants

The study sample was adults aged 16 years and over who took part in the nurse visit component of the HSE in 2003 or 2005 to 2014 . The study sample comprised adults who had a nurse visit; the analytical sample was adults who had observations for indoor temperature and self-rated health (Supplementary Figure S1). The analytic sample (n=74,736) was compared with those excluded from the study sample (n=2,065, Supplementary Table S1). There were no statistically significant differences between the analytic sample and those excluded regarding mean monthly outdoor temperatures by region (data not shown). However, among the categorical variables, only respiratory disease had non-significant differences in their distribution within the two samples. The analytic sample comprised 55% women, while 74% of the excluded participants were women ($P<0{\cdot}01$). The excluded survey participants had a younger age profile than the analytic sample; were less likely to be in the top two income quintiles; were more likely to live in social housing; but were more likely to have degree level qualifications. The analytic sample

For peer review only - http://bmjopen.bmj.com/site/about/guidelines.xhtml

were more likely to own their homes, and to have a longstanding illness or cardiovascular condition.

Research ethics approval

Research ethics approval was obtained prior to each year's survey from the relevant research ethics committees. Participants gave informed consent at each stage of the survey. Ethical approval was not required for this specific study, which was conducted on non-identifiable, archived data held by the UK Data Service for widespread research use.

Data

General health

General health was a self-assessed variable, referred to as self-rated health. It was recorded at the interview stage, prior to the nurse visit. The participants selected one of the five answer options *"Very good, good, fair, bad or very bad?"* to the question: *"How is your health in general?"* The wording of this question, which has been used throughout the Health Survey for England series, is identical to that recommended by the WHO in its 1996 report on harmonisation of instruments in health interview surveys[31]; the ONS (the Office for National Statistics) in 2008[32]; and the UK Government Statistical Service harmonisation.[32] For this analysis, general health was dichotomised into 'good', which encompassed the categories very good and good, and 'poor', which included fair, bad and very bad. Fair was placed in the 'poor' category rather than the 'good' category as the distribution of general health is highly skewed: 74% of participants reported good or very good general health.

Ill health

The other self-reported health data used in these analyses included the presence of limiting or non-limiting longstanding illness; a respiratory condition; or a cardiovascular condition. Participants were first asked if they had any longstanding illness or condition. Those who responded positively were then asked if it limited their daily activities, and asked what the condition(s) were, to a maximum of six. These were then coded into ICD-10 chapters, from which responses from participants volunteering a respiratory condition (chapter J) and/or a cardiovascular condition (chapter I) were identified.

Demographic data

Gender and age were collected at the interview.

Socio-economic data

Three variables were used for socio-economic position. Education level was categorised as degree or equivalent, qualification below degree, or no qualification. Equivalised household income, which adjusts for the numbers of adults and of children in the household, was divided into quintiles. The National Statistics-Socio-Economic Classification of occupation in five categories (NS-SEC5)[33] was derived from detailed information about employment status, occupation, size of organisation, and managerial responsibility. The categories were managerial & professional, intermediate, small employers, lower supervisory & technical, and semi-routine.

For peer review only - http://bmjopen.bmj.com/site/about/guidelines.xhtml

Housing

Housing information comprised the number of occupants in the household, and housing tenure. The latter was categorised as owner-occupied (e.g. owned outright or with a mortgage), rented privately, or socially rented (i.e. from a housing association or local government).

Temperature

Indoor air temperature was measured by the nurse, using a digital thermometer with a probe, prior to measuring blood pressure. The thermometer was placed on a surface near the blood pressure equipment, away from a radiator and out of direct sunlight. Nurses were recommended that the probe hang over the edge of the table.

Mean monthly outdoor temperature data by region were obtained from the Met Office website. The data are publicly available. The Met Office is a governmental agency in the UK which provides weather forecasts and warnings. The data were measured according to their standard protocol at weather stations 40 kilometres apart across the country, every hour.

Data sharing

The pseudonymised HSE data from each survey are deposited in the UK Data Archive and are available to be downloaded from the UK Data Service at: beta.ukdataservice.ac.uk/datacatalogue/series/series?id=2000021.

Statistical analysis

For the variables NS-SEC5, income and tenure, answers in the 'other' category, or categories outside of the main analytical categories (e.g. unemployed, student, or homemaker for NS-SEC) were grouped into an additional category of 'other or missing' and included in the models. This was because there were many participants in those categories and excluding them would have substantially decreased the sample size. The data were combined into a single dataset.

The analysis was run in Stata v15 (StataCorp, 2017). Models were built to test each hypothesis. Each model used the same analytical sample. The models were built using maximum likelihood estimation, and the best models were selected using the likelihood ratio test. Age was grouped for analysis as 16-24 then 10y groups to 75+, to avoid prior assumptions of the shape of the relationship.

Associations between socioeconomic and housing variables and indoor temperature were explored in two ways. Logistic regression models were created with indoor temperature <18℃ as the outcome variable (compared with indoor temperature of 18℃ or above) as well as linear regression models with indoor temperature as a continuous variable. For both sets of models, socioeconomic and housing variables were included as explanatory variables. Missing data among the covariates were included in the regression as an additional category; results are not shown in the tables.

To test the association of indoor temperature with general health, a similar set of logistic regression models were created but with indoor temperature as the explanatory variable, and general self-reported health as the outcome variable, again adjusting for demographic, housing, and socio-economic variables. The models were run for the whole sample, then

For peer review only - http://bmjopen.bmj.com/site/about/guidelines.xhtml

were then repeated stratified by tertile of outdoor temperature to assess if the association differed across the range of outdoor temperatures. As a sensitivity analysis, model 9 (fully adjusted) was repeated stratified by tertile of indoor temperature to assess if the association differed across the range of indoor temperatures.

The unadjusted coefficients of the variables are reported, as well as the coefficients after adjusting for other socioeconomic and housing variables. Collinearity was assessed by looking for reversal of the sign of the coefficients, and the variance inflation factor.

The analysis was weighted using the survey-provided weights, which account for probabilities of selection (due to the sample design), and response rate differences by age, sex, and region. This ensured a weighted sample which matched the ONS-mid-year population estimates by age and sex for each survey year.[30] The multi-stage survey design was accounted for using the 'svy' commands in STATA, using the survey's primary sampling units, and using government office region as the strata variable, since this was a component of stratification that was consistent across the survey years.

Results

The analytical sample consisted of 74,736 participants, 97·3% of the study sample. Their characteristics are shown in Supplementary Table S1. The indoor temperature ranged from 7·5°C to 36·8°C, with a mean of 20·7°C (SD 2·3). The outdoor temperature ranged from -1·2°C to 20·5°C, with a mean of 10·1°C (SD 4·6). For both indoor and outdoor temperature, most of the variation was attributable to month. 10% of households had indoor temperatures below the UK government's recommended minimum of 18°C.

<Insert Table 1 around here>

Socioeconomic and housing variables and indoor temperature

Households with more occupants and higher equivalised household incomes were associated with a lower odds of an indoor temperature <18°C (Table 1) and higher mean indoor temperatures (Tables 2 and S1). Higher educational attainment, and private sector rented houses were associated with lower mean indoor temperatures (Table 2) and a higher odds of the indoor temperature being <18°C (Table 1). NS-SEC5 was variably associated with low temperature (reduced odds among intermediate but higher odds among small employers) and with mean indoor temperature (positive association in lower supervisory and technical households but inverse association among small employers) in the fully adjusted models, compared with those in managerial and professional occupations.

<Insert Table 2 around here>

For peer review only

Table 1 - Logistic regression models of associations of socioeconomic and housing variables with low indoor temperature (<18°C)

	Minimally adjusted[a]				Fully adjusted[b]			
	OR	95% CI		P	OR	95% CI		P
**Household size**								
1	1				1			
2	0·58	0·54	0·63	<0·001	0·59	0·54	0·63	<0·001
3	0·46	0·41	0·51	<0·001	0·46	0·41	0·51	<0·001
4	0·38	0·34	0·43	<0·001	0·38	0·34	0·43	<0·001
5+	0·37	0·32	0·44	<0·001	0·36	0·31	0·43	<0·001
**Tenure**								
Owner-occupied	1				1			
Privately rented	1·49	1·33	1·68	<0·001	1·27	1·12	1·43	<0·001
Socially rented	1·10	1·00	1·20	0·056	0·94	0·85	1·05	0·264
**NS-SEC5**								
Managerial & professional	1				1			
Intermediate	0·90	0·83	0·98	0·015	0·92	0·84	1·00	0·047
Small employers	1·17	1·06	1·29	0·003	1·20	1·09	1·33	<0·001
Lower supervisory & technical	0·91	0·81	1·01	0·083	0·93	0·83	1·05	0·236
Semi-routine	1·00	0·94	1·08	0·901	1·02	0·94	1·11	0·585
**Education**								
Degree	1				1			
Qualification below degree	0·91	0·85	0·98	0·017	0·92	0·85	1·00	0·045
No qualification	0·90	0·82	0·99	0·029	0·84	0·75	0·93	0·001
**Equivalised household income quintile**								
Lowest quintile	1				1			
2nd quintile	0·87	0·78	0·98	0·024	0·91	0·81	1·03	0·126
3rd quintile	0·86	0·77	0·96	0·010	0·88	0·78	0·99	0·030
4th quintile	0·84	0·75	0·95	0·004	0·83	0·73	0·94	0·003
Highest quintile	0·86	0·77	0·97	0·013	0·77	0·67	0·88	<0·001

[a] Minimally adjusted models: adjusted for outdoor temperature and age group.

[b] Fully adjusted models: mutually adjusted for all socioeconomic and housing variables in this table.

For peer review only

For peer review only - http://bmjopen.bmj.com/site/about/guidelines.xhtml

Table 2 - Linear regression models with socioeconomic and housing variables as explanatory variables for indoor temperature

	Minimally adjusted[a]				Fully adjusted[b]			
	β	95% CI		P	β	95% CI		P
**Household size**								
1	Ref							
2	0·41	0·35	0·46	<0·001	0·43	0·38	0·49	<0·001
3	0·63	0·56	0·71	<0·001	0·65	0·58	0·73	<0·001
4	0·74	0·66	0·82	<0·001	0·76	0·68	0·85	<0·001
5+	0·79	0·68	0·90	<0·001	0·81	0·70	0·93	<0·001
**Tenure**								
Owner-occupied	Ref							
Privately rented	-0·23	-0·32	-0·14	<0·001	-0·11	-0·20	-0·02	0·017
Socially rented	0·13	0·06	0·19	<0·001	0·21	0·14	0·28	<0·001
**NS-SEC5**								
Managerial and professional occupations	Ref							
Intermediate	0·08	0·03	0·13	0·003	0·03	-0·03	0·08	0·298
Small employers	-0·05	-0·12	0·02	0·177	-0·11	-0·18	-0·04	0·001
Lower supervisory and technical	0·18	0·11	0·24	<0·001	0·09	0·02	0·16	0·010
Semi-routine	0·06	0·01	0·11	0·020	-0·04	-0·09	0·01	0·128
**Education**								
Degree	Ref							
Qualification below degree	0·16	0·11	0·21	<0·001	0·14	0·08	0·19	<0·001
No qualification	0·22	0·16	0·29	<0·001	0·22	0·15	0·29	<0·001
**Income quintile**								
Lowest quintile	Ref							
2nd quintile	0·03	-0·05	0·11	0·468	0·04	-0·05	0·12	0·402
3rd quintile	0·02	-0·06	0·10	0·631	0·07	-0·01	0·16	0·081
4th quintile	0·01	-0·07	0·09	0·843	0·11	0·02	0·19	0·016
Highest quintile	-0·04	-0·12	0·05	0·402	0·15	0·06	0·24	0·001

[a] Minimally adjusted models: adjusted for outdoor temperature and age group.

[b] Fully adjusted models: mutually adjusted for all socioeconomic and housing variables in this table.

For peer review only

Indoor temperature and health

Each one degree increase in indoor temperature was associated with a 1.4% (95% CI 0.7-2.6) increase in odds of poor health, after adjusting for age and gender (see Figure 1). After adjusting for socioeconomic and housing factors, each degree increase in temperature is associated with a 1.7% (95% CI 0.7-2.6) increase in odds of poor health.

<Insert Figure 1 around here>

A series of regression models were run to examine the impact on the odds of reporting poor health associated with indoor temperature of demographic, housing, and socio-economic factors (Table 3). Adjusting for household size increased the odds ratio most, with small changes for household size and tenure or for equivalised household income . Adjusting for tenure, NS-SEC5, or all three socio-economic factors reduced the odds ratio a little. The linear association between indoor temperature and reporting poor health remained significant in each model; the final model, including all these covariates, also increased the odds ratio compared with the base model.

When stratified by indoor temperature range, and adjusting for socioeconomic and housing factors, the odds ratios for each tertile did not differ significantly from one another (sensitivity analysis, data not shown). In addition, with the smaller sample size and truncated range of temperatures, the relationship between indoor temperature and poor health was no longer significant within any of the tertiles.

<Insert Table 3 around here>

When regression models were repeated stratified for tertile of outdoor temperature, we found the ORs of indoor temperature on poor health were stronger for the coldest tertile (i.e. <7˚C). The point estimates for the warmer tertiles of outdoor temperature were similar to the overall ORs but were not significantly above unity.

For peer review only - http://bmjopen.bmj.com/site/about/guidelines.xhtml

Table 3 - Logistic regression models showing odds ratios of poor health for indoor temperature

Indoor temperature[a,b]	All				Lowest tertile of outdoor temperature (<7˚C)				Middle tertile of outdoor temperature (7-12.8˚C)				Highest tertile of outdoor temperature (≥12.9˚C)			
	**OR**	**95% CI**		**P**	**OR**	**95% CI**		**P**	**OR**	**95% CI**		**P**	**OR**	**95% CI**		**P**
Model 1: adjusted for age and gender	1·01	1·00	1·02	0·003	1.02	1.01	1.04	0.004	1.01	1.00	1.03	0.122	1.02	1.00	1.03	0.069
Model 2: Model 1 + Household size	1·02	1·01	1·03	<0·001	1.04	1.02	1.05	<0·001	1.02	1.00	1.04	0.018	1.02	1.00	1.03	0.032
Model 3: Model 1 + Tenure	1·01	1·00	1·02	0·014	1.03	1.01	1.05	0.001	1.01	0.99	1.03	0.275	1.00	0.99	1.02	0.614
Model 4: Model 1 + NS-SEC5	1·01	1·00	1·02	<0·005	1.02	1.01	1.04	0.007	1.02	1.00	1.03	0.091	1.01	1.00	1.03	0.163
Model 5: Model 1 + Income	1·02	1·01	1·03	<0·001	1.03	1.01	1.05	0.001	1.01	1.00	1.03	0.138	1.01	1.00	1.03	0.117
Model 6: Model 1 + Education	1·01	1·00	1·02	0·034	1.02	1.00	1.03	0.029	1.01	0.99	1.03	0.276	1.01	0.99	1.03	0.208
Model 7: Model 1 + housing variables	1·02	1·01	1·02	0·002	1.04	1.02	1.05	<0·001	1.01	1.00	1.03	0.132	1.01	0.99	1.02	0.47
Model 8, Model 1 + socioeconomic variables	1·01	1·00	1·02	0·007	1.02	1.01	1.04	0.004	1.01	0.99	1.03	0.191	1.01	0.99	1.03	0.31
Model 9: Model 1 + housing and socioeconomic variables	1·02	1·01	1·03	<0·001	1.04	1.02	1.05	<0·001	1.02	1.00	1.04	0.086	1.01	0.99	1.02	0.465

[a] Indoor temperature was linear in these models.
[b] Models are listed in order of increasing likelihood. All models adjusted for age group and gender; Model 7 as Model 1 plus household size and tenure; Model 8 as Model 1 plus NS-SEC5, equivalised income quintiles, and education; Model 9 included all the variables.

For peer review only

Discussion

The results showed an association between higher indoor temperatures and increased odds of poor self-rated health. This has also been shown recently in a small number of individuals in Cornwall, UK: participants with poorer health maintained their homes at higher temperatures.[34] However, the relationship between socioeconomic and housing factors with indoor temperature was complicated, with different measures having different directions of association with indoor temperature (Figure 2). The most intuitive association is that those with lower incomes tended to have lower indoor temperatures, possibly because they cannot afford to pay for gas or electricity to heat their homes. This may explain why the association between indoor temperature and poor health was strongest when the outdoor temperature was lower. Conversely, other markers of lower social class, including lower NS-SEC5 and lower education, were associated with warmer indoor temperatures. These differing associations might be best explained by the finding that privately rented accommodation was cooler than homes that were socially rented. This is a reflection that the ability to raise indoor temperature is determined by both the financial ability to pay for fuel and the insulation of the home. Socially rented accommodation tends to be better insulated than privately rented accommodation both because of the housing quality and space standards required on social housing[35] and because social landlords such as housing associations and local councils may have more incentive to provide decent quality accommodation as a longterm asset. A small study in Japan, using perceived thermal comfort not measured temperature, found that people who reported living in cold homes had a higher risk of frailty - but only among those who were dissatisfied with their economic situation.[36]

It is not clear from this research why temperatures are higher among households with worse self-rated health and whether this is due to personal preferences and behaviours; advice from medical or other professionals to maintain a warmer home; the difference in the type of building they occupy (i.e. dwellings that are typically warmer such as smaller houses or flats, and social housing rather than privately rented); or some combination of these. Figure 2 shows a conceptual framework for the way in which these variables affect the associations.

<Figure 2 around here>

The strengths of this study are the large number of participants, who were nationally-representative of the non-institutionalised population living in England. The temperature measurements were taken using a standard protocol across the time period but were not the focus of the surveys, so the likelihood of differential response rates systematically associated with indoor temperature is very small. Unlike the study by Armstrong and colleagues, we report data based on individuals, rather than ecological analyses.[37]

Limitations include the significant differences in many characteristics between the analytic sample, who had indoor measurements, and those excluded because there were no measurements. The latter comprises those who were ineligible for blood pressure measurement (for example being pregnant) or declined the measurement. However, fewer than 3% of the study sample were excluded; the analytic sample is therefore almost identical to the study sample overall. The other main limitation was the measurement of indoor temperature at a single time point for each participant in whichever room they were 'hosting' the survey nurse, generally in the living room or kitchen. Huebner and colleagues have

For peer review only - http://bmjopen.bmj.com/site/about/guidelines.xhtml

shown four different profiles in living room temperature variability across the day, varying from bimodal (morning and evening peaks), steadily increasing (shallow and steep) to flat with little variation.[38]

Hajat and colleague have shown that 40% of cold-related deaths occur outside the December-March period used for calculating 'excess winter deaths'.[27] For that reason, and given seasonal and temperature differences across the regions of England, we used tertile of outdoor temperature rather than season of other groupings of months. We showed a strong relationship at low outdoor temperatures but little if any effect at high outdoor temperatures. Impacts of heatwaves are generally acute, so it is less likely that we would have found a strong relationship at higher outdoor temperatures.

While the subjectivity of self-rated health makes it vulnerable to information bias, it has been shown to be highly predictive of future mortality and health outcomes, and is used frequently across the scientific literature.[39,40] Self-rated health is strongly associated with total mortality [41–45] and with some specific causes of death[46,47]but not with external causes.[46] It also predicts functional ability.[48] Self-rated health is explained more by measures of mental and physical health than by demographic or socio-economic factors.[49]

Apart from the well-known association between temperature and blood pressure,[50–53] there is less evidence pointing at more specific health outcomes related to temperature. Thus, as this is the first study of its kind, the broadness of general health as the outcome was a strength rather than a weakness. The differences between the entire sample and those with complete data on self-rated health and temperature was primarily due to non-response to the nurse visit among interviewees. This was dealt with by use of non-response weighting.[29]

While guidelines on healthy indoor temperatures have been issued by the World Health Organisation (WHO) and the UK Public Health England, they appear arbitrary and the WHO itself describe its own temperature recommendations as *'[scientifically] weak and not well-known to the public or policy makers'*.[54–56] Since 2014, the recommendation from the UK government is for a minimum of 18°C in all rooms, a change from the previous 21°C for living rooms.[57] The government attributed the change to a review of evidence, awareness of climate change and financial difficulties, and acceptability (*"We know that people have strong feelings about their homes and don't want to be told what to do in them")*.[56] Further research is needed to understand the temporality and mechanism of the association.

A recent study in England found no impact of Winter Fuel Payment eligibility on indoor temperature,[58] but no study has examined at individual level the impacts of energy efficiency retrofits on indoor temperature, residents' heating-relate behaviours, or health. When linked data are available, we intend to examine the associations of indoor temperature with subsequent hospital admissions and mortality. This will be important for assessing and making health-related policy recommendations for indoor temperature. Future research in this area could take advantage of internet-enabled 'smart' thermostats.

For peer review only - http://bmjopen.bmj.com/site/about/guidelines.xhtml

Figure captions

Figure 1 - Predicted prevalence of poor health for each degree increase in indoor temperature

Footnote for Figure 1:

Prevalence of poor health predicted by the logistic regression of indoor temperature on poor health, adjusted for age and sex. OR 1.01 (95% CI 1.00-1.02), see Table 3, model 1.

Figure 2 - Conceptual framework for temperature-health association

For peer review only

For peer review only - http://bmjopen.bmj.com/site/about/guidelines.xhtml

Acknowledgements

We thank the nurses and participants in the Health Surveys for England, and our colleagues at NatCen Social Research and UCL.

Author contributions

This work was conducted as part of JS-K's MSc in Social Epidemiology. She led on conducting the literature search, designing and conducting the analyses, creating the figures, and interpreting the data. AM and JM contributed advice to the dissertation. JS-K and JM wrote the initial draft of the manuscript. IH updated the literature search and contributed to analysis and preparation of the manuscript. All authors contributed to subsequent drafts, approved the final manuscript, and are accountable for the accuracy and integrity of all aspects of the work.

Patient and Public Involvement

This study does not involve patients as the data come from a cross-section of the general population, regardless of their involvement (or not) with any health services. The public were not involved in the design of this study but the Health Survey for England itself has an Advisory Group that includes stakeholders from local government, public health, the Department of Health and Social Care, Public Health England, NHS Digital, and academe.

Age UK, Kilburn Older Voices (KOVE), the Department of Business, Energy and Industrial Strategy (BEIS), the Met Office, NatCen Social Research, and Public Health England will help with the dissemination of these findings.

Role of the funding source

NHS Digital, which funds the HSE, also funds AM & JM to work on the annual HSE. This work was also supported by the EPSRC [EP/R035288/1] to fund IH to work on home energy efficiency. NHS Digital and EPSRC played no part in the planning, conduct, or interpretation of these analyses; the drafting of the manuscript; nor the decision to publish.

Conflict of interest statement

Professor Mindell and Ms. Moody report grants from NHS Digital during the conduct of the study; Dr. Sutton-Klein has nothing to disclose. Dr. Hamilton report grants from the Engineering and Physical Sciences Research Council (EPSRC).

For peer review only

References

1 Analitis A, Katsouyanni K, Biggeri A, *et al.* Effects of Cold Weather on Mortality: Results From 15 European Cities Within the PHEWE Project. *American Journal of Epidemiology* 2008;**168**:1397–408. doi:10.1093/aje/kwn266

2 Tham S, Thompson R, Landeg O, *et al.* Indoor temperature and health: a global systematic review. *Public Health* 2019;**179**:9–17. doi:10.1016/j.puhe.2019.09.005

3 Marmot Review Team. the health impacts of cold homes and fuel poverty. London: : Friends of the Earth 2011. http://www.instituteofhealthequity.org/resources-reports/the-health-impacts-of-cold-homes-and-fuel-poverty/the-health-impacts-of-cold-homes-and-fuel-poverty.pdf (accessed 14 Jul 2020).

4 Hammel HT, Pierce JB. Regulation of Internal Body Temperature. *Annu Rev Physiol* 1968;**30**:641–710. doi:10.1146/annurev.ph.30.030168.003233

5 Mallet ML. Pathophysiology of accidental hypothermia. *QJM* 2002;**95**:775–85. doi:10.1093/qjmed/95.12.775

6 ONS. Excess Winter Mortality in England and Wales : 2018/19 (Provisional) and 2017/18 (Final). ONS 2019. https://www.ons.gov.uk/peoplepopulationandcommunity/birthsdeathsandmarriages/deaths/bulletins/excesswintermortalityinenglandandwales/2018to2019provisionaland2017to2018final

7 Liddell C, Morris C, Thomson H, *et al.* Excess winter deaths in 30 European countries 1980–2013: a critical review of methods. *J Public Health* 2015;:fdv184. doi:10.1093/pubmed/fdv184

8 Laake K, Sverre JM. Winter Excess Mortality: A Comparison between Norway and England plus Wales. *Age Ageing* 1996;**25**:343–8. doi:10.1093/ageing/25.5.343

9 Fowler T, Southgate RJ, Waite T, *et al.* Excess Winter Deaths in Europe: a multi-country descriptive analysis. *European Journal of Public Health* 2015;**25**:339–45. doi:10.1093/eurpub/cku073

10 Healy JD. Excess winter mortality in Europe: a cross country analysis identifying key risk factors. *Journal of Epidemiology & Community Health* 2003;**57**:784–9. doi:10.1136/jech.57.10.784

11 Collins KJ. Low indoor temperatures and morbidity in the elderly. *Age Ageing* 1986;**15**:212–20. doi:10.1093/ageing/15.4.212

12 Clinch JP. Housing standards and excess winter mortality. *Journal of Epidemiology & Community Health* 2000;**54**:719–20. doi:10.1136/jech.54.9.719

13 Howden-Chapman P, Matheson A, Crane J, *et al.* Effect of insulating existing houses on health inequality: cluster randomised study in the community. *BMJ* 2007;**334**:460. doi:10.1136/bmj.39070.573032.80

14 Lloyd EL, McCormack C, McKeever M, *et al.* The effect of improving the thermal quality of cold housing on blood pressure and general health: a research note. *Journal of Epidemiology & Community Health* 2008;**62**:793–7. doi:10.1136/jech.2007.067835

For peer review only - http://bmjopen.bmj.com/site/about/guidelines.xhtml

15 Ormandy D, Ezratty V. Health and thermal comfort: From WHO guidance to housing strategies. *Energy Policy* 2012;**49**:116–21. doi:10.1016/j.enpol.2011.09.003

16 Evans J. An epidemiological study of the relative importance of damp housing in relation to adult health. *Journal of Epidemiology & Community Health* 2000;**54**:677–86. doi:10.1136/jech.54.9.677

17 Wilkinson P, Landon M, Armstrong B, *et al.* Cold comfort: the social and environmental determinants of excess winter death in England, 1986-1996. York: : Joseph Rowntree Foundation 2001. https://researchonline.lshtm.ac.uk/id/eprint/17944

18 Hamilton IG, O'Sullivan A, Huebner G, *et al.* Old and cold? Findings on the determinants of indoor temperatures in English dwellings during cold conditions. *Energy and Buildings* 2017;**141**:142–57. doi:10.1016/j.enbuild.2017.02.014

19 Jevons R, Carmichael C, Crossley A, *et al.* Minimum indoor temperature threshold recommendations for English homes in winter – A systematic review. *Public Health* 2016;**136**:4–12. doi:10.1016/j.puhe.2016.02.007

20 Huebner GM, Hamilton I, Chalabi Z, *et al.* Comparison of indoor temperatures of homes with recommended temperatures and effects of disability and age: an observational, cross-sectional study. *BMJ Open* 2018;**8**:e021085. doi:10.1136/bmjopen-2017-021085

21 Lelkes O, Zolyomi E. Quality of housing and the link to income. Brussels: : European Commission 2009.

22 Tunstall R, Bevan M, Bradshaw J, *et al.* The links between housing and poverty: an evidence review. York: : Joseph Rowntree Foundation 2013.

23 Marshall C, Moody A, Hamilton I, *et al.* Changes in heating of homes from 1991 to 2011 using data from the ONS-Longitudinal Study.

24 Ministry of Housing, Communities and Local Government. English Housing Survey 2016 to 2017: headline report. London: : MHCLG 2018.

25 Oreszczyn T, Hong SH, Ridley I, *et al.* Determinants of winter indoor temperatures in low income households in England. *Energy and Buildings* 2006;**38**:245–52. doi:10.1016/j.enbuild.2005.06.006

26 Public Health England. PHE heatwave mortality monitoring, Summer 2019. London: : PHE https://assets.publishing.service.gov.uk/government/uploads/system/uploads/attachment_data/file/841320/PHE_heatwave_report_2019.pdf (accessed 24 Jul 2020).

27 Hajat S, Gasparrini A. The Excess Winter Deaths Measure: Why Its Use Is Misleading for Public Health Understanding of Cold-related Health Impacts. *Epidemiology* 2016;**27**:486–91. doi:10.1097/EDE.0000000000000479

28 Mindell J, Biddulph JP, Hirani V, *et al.* Cohort Profile: The Health Survey for England. *International Journal of Epidemiology* 2012;**41**:1585–93. doi:10.1093/ije/dyr199

29 Mindell JS, Goesswald A, Kamtsiuris P, *et al.* Sample selection, recruitment and participation rates in health examination surveys in Europe – experience from seven national surveys. *BMC Med Res Methodol* 2015;**15**:78. doi:10.1186/s12874-015-0072-4

For peer review only

For peer review only - http://bmjopen.bmj.com/site/about/guidelines.xhtml

30 Craig R, Fuller E, Mindell J (Eds.). Health Survey for England 2014. Volume 2. Methods and documentation. Leeds: : NHS Digital 2015. https://digital.nhs.uk/data-and-information/publications/statistical/health-survey-for-england/health-survey-for-england-2014

31 de Bruin A, Picavet HSJ, Nossikov A, editors. *Health interview surveys: towards international harmonization of methods and instruments*. Copenhagen: : World Health Organization, Regional Office for Europe 1996. https://www.euro.who.int/__data/assets/pdf_file/0017/111149/E72841.pdf?ua=1 (accessed 14 Jul 2020).

32 Nixon O. General health harmonised principle. Government Statistical Service. 2020.https://gss.civilservice.gov.uk/policy-store/general-health/ (accessed 14 Jul 2020).

33 The National Statistics Socio-economic classification (NS-SEC). Office for National Statistics. 2016.https://www.ons.gov.uk/methodology/classificationsandstandards/otherclassifications/thenationalstatisticssocioeconomicclassificationnssecrebasedonsoc2010 (accessed 15 Jul 2020).

34 Pollard A, Jones T, Sherratt S, *et al.* Use of Simple Telemetry to Reduce the Health Impacts of Fuel Poverty and Living in Cold Homes. *Int J Environ Res Public Health* 2019;**16**. doi:10.3390/ijerph16162853

35 Park J. One Hundred Years of Housing space Standards. what now?Housing Space Standards. London: : Levitt Bernstein 2017. https://levittbernstein.co.uk/site/assets/files/2682/one_hundred_years_of_space_standards.pdf

36 Nakajima Y, Schmidt SM, Malmgren Fänge A, *et al.* Relationship between Perceived Indoor Temperature and Self-Reported Risk for Frailty among Community-Dwelling Older People. *Int J Environ Res Public Health* 2019;**16**. doi:10.3390/ijerph16040613

37 Armstrong B, Bonnington O, Chalabi Z, *et al. The impact of home energy efficiency interventions and winter fuel payments on winter- and cold-related mortality and morbidity in England: a natural equipment mixed-methods study*. Southampton (UK): : NIHR Journals Library 2018. http://www.ncbi.nlm.nih.gov/books/NBK532137/ (accessed 3 Dec 2019).

38 Huebner GM, McMichael M, Shipworth D, *et al.* The shape of warmth: temperature profiles in living rooms. *Building Research & Information* 2015;**43**:185–96. doi:10.1080/09613218.2014.922339

39 Simon JG, De Boer JB, Joung IMA, *et al.* How is your health in general? A qualitative study on self-assessed health. *European Journal of Public Health* 2005;**15**:200–8. doi:10.1093/eurpub/cki102

40 Jylhä M. What is self-rated health and why does it predict mortality? Towards a unified conceptual model. *Social Science & Medicine* 2009;**69**:307–16. doi:10.1016/j.socscimed.2009.05.013

41 Heistaro S, Jousilahti P, Lahelma E, *et al.* Self rated health and mortality: a long term prospective study in eastern Finland. *Journal of Epidemiology & Community Health* 2001;**55**:227–32. doi:10.1136/jech.55.4.227

For peer review only

For peer review only - http://bmjopen.bmj.com/site/about/guidelines.xhtml

42 Idler EL, Benyamini Y. Self-rated health and mortality: a review of twenty-seven community studies. *J Health Soc Behav* 1997;**38**:21–37.

43 Kaplan GA, Camacho T. Perceived health and mortality: A nine-year follow-up of the Human Population Laboratory Cohort. *American Journal of Epidemiology* 1983;**117**:292–304. doi:10.1093/oxfordjournals.aje.a113541

44 Møller L, Kristensen TS, Hollnagel H. Self rated health as a predictor of coronary heart disease in Copenhagen, Denmark. *Journal of Epidemiology & Community Health* 1996;**50**:423–8. doi:10.1136/jech.50.4.423

45 Mossey JM, Shapiro E. Self-rated health: a predictor of mortality among the elderly. *Am J Public Health* 1982;**72**:800–8. doi:10.2105/AJPH.72.8.800

46 Benjamins MR, Hummer RA, Eberstein IW, *et al.* Self-reported health and adult mortality risk: An analysis of cause-specific mortality. *Social Science & Medicine* 2004;**59**:1297–306. doi:10.1016/j.socscimed.2003.01.001

47 Wannamethee G, Shaper AG. Self-assessment of Health Status and Mortality in Middle-Aged British Men. *Int J Epidemiol* 1991;**20**:239–45. doi:10.1093/ije/20.1.239

48 Idler EL, Kasl SV. Self-Ratings of Health: Do they also Predict change in Functional Ability? *The Journals of Gerontology Series B: Psychological Sciences and Social Sciences* 1995;**50B**:S344–53. doi:10.1093/geronb/50B.6.S344

49 Singh-Manoux A. What does self rated health measure? Results from the British Whitehall II and French Gazel cohort studies. *Journal of Epidemiology & Community Health* 2006;**60**:364–72. doi:10.1136/jech.2005.039883

50 Umishio W, Ikaga T, Kario K, *et al.* Cross-Sectional Analysis of the Relationship Between Home Blood Pressure and Indoor Temperature in Winter: A Nationwide Smart Wellness Housing Survey in Japan. *Hypertension* 2019;**74**:756–66. doi:10.1161/HYPERTENSIONAHA.119.12914

51 Wang Q, Li C, Guo Y, *et al.* Environmental ambient temperature and blood pressure in adults: A systematic review and meta-analysis. *Science of The Total Environment* 2017;**575**:276–86. doi:10.1016/j.scitotenv.2016.10.019

52 Zhao H, Jivraj S, Moody A. “My blood pressure is low today, do you have the heating on?” The association between indoor temperature and blood pressure. *J Hypertens* 2019;**37**:504–12. doi:10.1097/HJH.0000000000001924

53 Cabrera SE, Mindell JS, Toledo M, *et al.* Associations of Blood Pressure With Geographical Latitude, Solar Radiation, and Ambient Temperature: Results From the Chilean Health Survey, 2009–2010: Table 1. *Am J Epidemiol* 2016;**183**:1071–3. doi:10.1093/aje/kww037

54 World Health Organization Regional Office for Europe. Housing, energy and thermal comfort : a review of 10 countries within the WHO European Region. Copenhagen: : WHO Regional Office for Europe 2007. https://apps.who.int/iris/handle/10665/107815

55 World Health Organization. The Effects of the Indoor Housing Climate on the Health of the Elderly: Report on a WHO Working Group, Graz, Austria, 20-24 September 1982. Copenhagen: : WHO Regional Office for Europe 1984.

For peer review only - http://bmjopen.bmj.com/site/about/guidelines.xhtml

56 Bone D, Carmichael K, Wookey R. The Cold Weather Plan for England: Protecting health and reducing harm from cold weather. London: : Public Health England 2014.

57 Wookey R, Bone A, Carmichael C, *et al.* Minimum home temperature thresholds for health in winter – A systematic literature review. London: : Public Health England 2014.

58 Angelini V, Daly M, Moro M, *et al. The effect of the Winter Fuel Payment on household temperature and health: a regression discontinuity design study*. Southampton (UK): : NIHR Journals Library 2019. http://www.ncbi.nlm.nih.gov/books/NBK535694/ (accessed 3 Dec 2019).

For peer review only

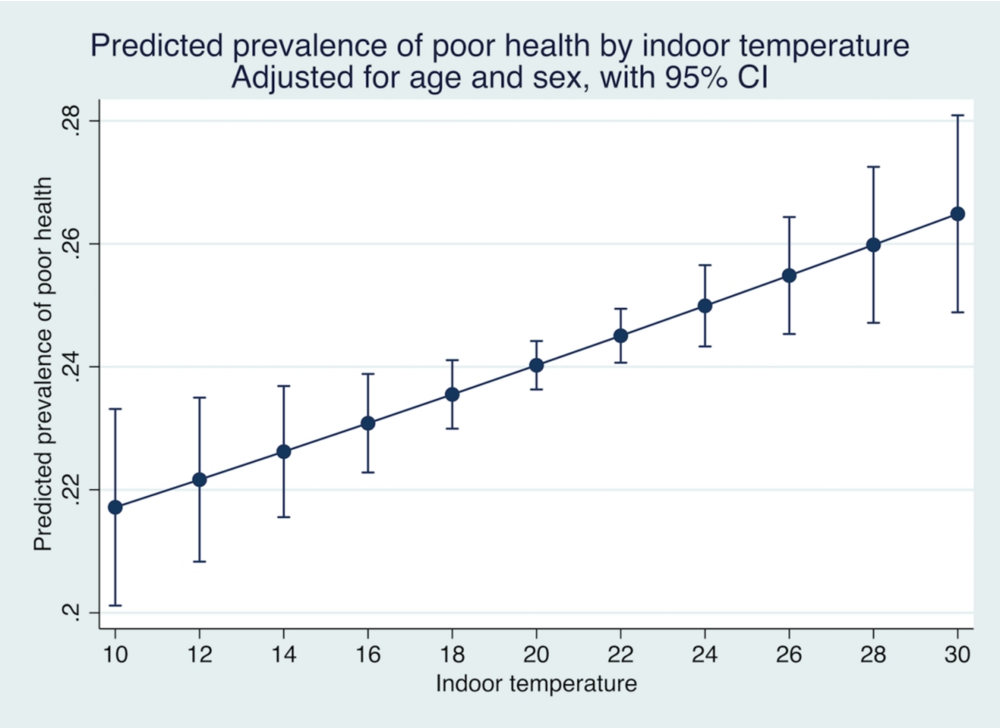

Caption: Predicted prevalence of poor health for each degree increase in indoor temperature
Legend: Prevalence of poor health predicted by the logistic regression of indoor temperature on poor health, adjusted for age and sex. OR 1.01 (95% CI 1.00-1.02), see Table 3, model 1.

123x90mm (300 x 300 DPI)

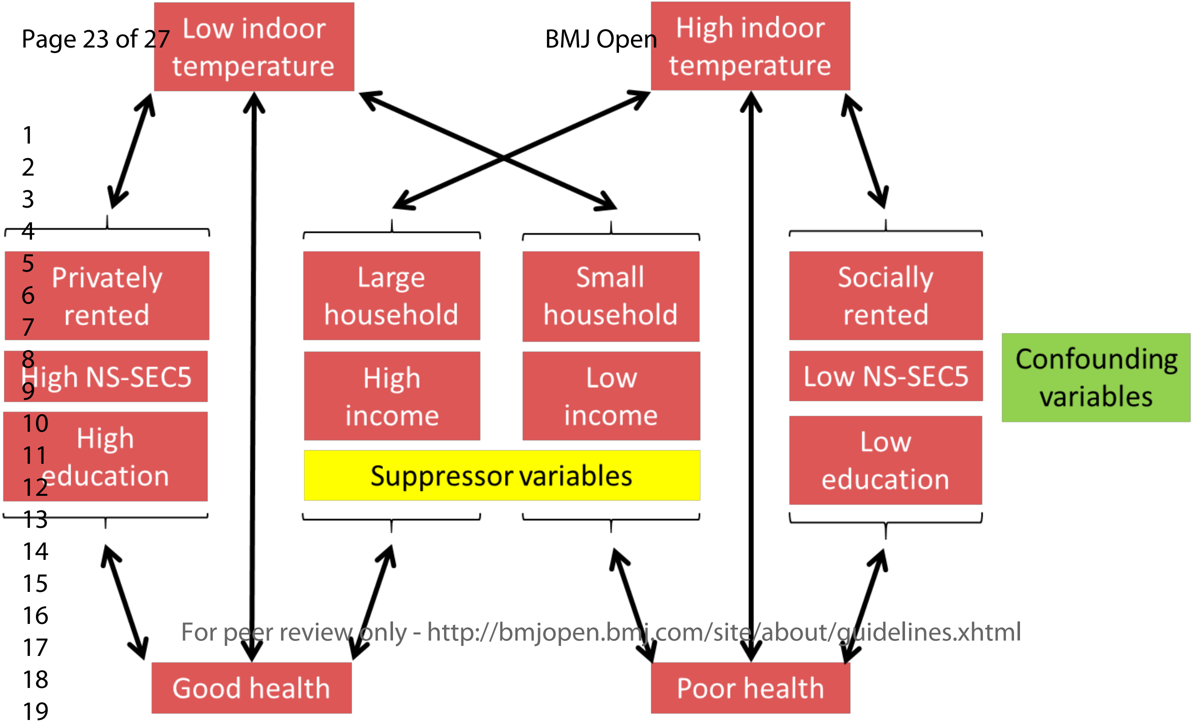

For peer review only - http://bmjopen.bmj.com/site/about/guidelines.xhtml

Supplementary material

Table S1. Characteristics of participants

Variable	Category	Analytical sample[a] N*	%	Excluded from analysis[b] N	%	Study Sample[c] N	%
Gender[d]	Men	33,526	45%	533	26%	34,059	44%
	Women	41,210	55%	1,532	74%	42,742	56%
Age group[d]	16-24	6,593	9%	373	18%	6,966	9%
	25-34	9,589	13%	639	31%	10,228	13%
	35-44	13,145	18%	375	18%	13,520	18%
	45-54	12,929	17%	179	9%	13,108	17%
	55-64	12,753	17%	161	8%	12,914	17%
	65-74	11,315	15%	162	8%	11,477	15%
	75+	8,412	11%	176	9%	8,588	11%
NS-SEC5[d]	Managerial/ professional	25,297	34%	557	27%	25,854	34%
	Intermediate Occupation	10,354	14%	234	11%	10,588	14%
	Small employers	6,514	9%	91	4%	6,605	9%
	Lower supervisory	6,087	8%	96	5%	6,183	8%
	Semi-routine	23,314	31%	511	25%	23,825	31%
	Missing data	*3,170*	*4%*	132	6%	*3,302*	*4%*
Education[d]	Degree level or higher	15,400	21%	476	23%	15,876	21%
	Qualification below degree	40,233	54%	992	48%	41,225	54%
	No qualification	19,103	26%	515	25%	19,618	26%
	Missing data	*0*	*0%*	*82*	*4%*	*82*	*0.1%*
Income quintile[d]	Lowest	10,861	15%	420	20%	11,281	15%
	2nd lowest	12,115	16%	302	15%	12,417	16%
	3rd	13,190	18%	260	13%	13,450	18%
	4th	13,537	18%	265	13%	13,802	18%
	Highest	13,291	18%	321	16%	13,612	18%
	Missing data	*11,742*	*16%*	461	22%	*12,203*	*16%*
Tenure[d]	Owner-occupied	55,159	74%	1,076	52%	56,235	73%
	Privately rented	6,476	9%	283	14%	6,759	9%
	Socially rented	11,347	15%	492	24%	11,839	15%
	Missing data	*1,754*	*2%*	214	10%	*1,968*	*3%*
Household size[d]	1	13,677	18%	284	14%	13,961	18%
	2	30,117	40%	712	34%	30,829	40%
	3	12,585	17%	540	26%	13,125	17%
	4	12,173	16%	323	16%	12,496	16%
	5+	6,184	8%	206	10%	6,390	8%
Tertile of	Low	24,927	33%	653	32%	25,580	33%

For peer review only

regional mean outdoor temperature at month of visit[e]	Medium	24,506	33%	752	35%	25,238	33%
	High	25,303	34%	680	33%	25,983	34%
General health[f]	Good	55,235	74%	1,567	76%	56,802	74%
	Poor	19,501	26%	498	24%	19,999	26%
Longstanding illness[d]	No	39,246	53%	1,269	61%	40,515	53%
	Yes	35,490	47%	781	38%	36,271	47%
Heart condition[d]	No	63,905	86%	1,838	89%	65,743	86%
	Yes	10,831	14%	212	10%	11,043	14%
Respiratory condition[g]	No	68,051	91%	1,869	91%	69,920	91%
	Yes	6,685	9%	181	9%	6,866	9%
Total		*74,736*	*97.3%*	*2,065*	*2.7%*	*76,801*	*100%*

[a] Analytical sample was all participants with a nurse visit and with information on both self-rated health and indoor temperature'.

[b] Excluded sample are those HSE participants who had a nurse visit (so are included in the study sample) but had missing data for indoor temperature and/or self-rated health

[c] Study sample: all those with a nurse visit

[d] X^2 test for difference between analytic sample and excluded participants: p<0.001

[e] X^2 test for difference between analytic sample and excluded participants: p=0.036

[f] X^2 test for difference between analytic sample and excluded participants: p=0.043

[g] X^2 test for difference between analytic sample and excluded participants: p=0.858

For peer review only

Figure S1. Flow diagram showing selection of data into analytical sample

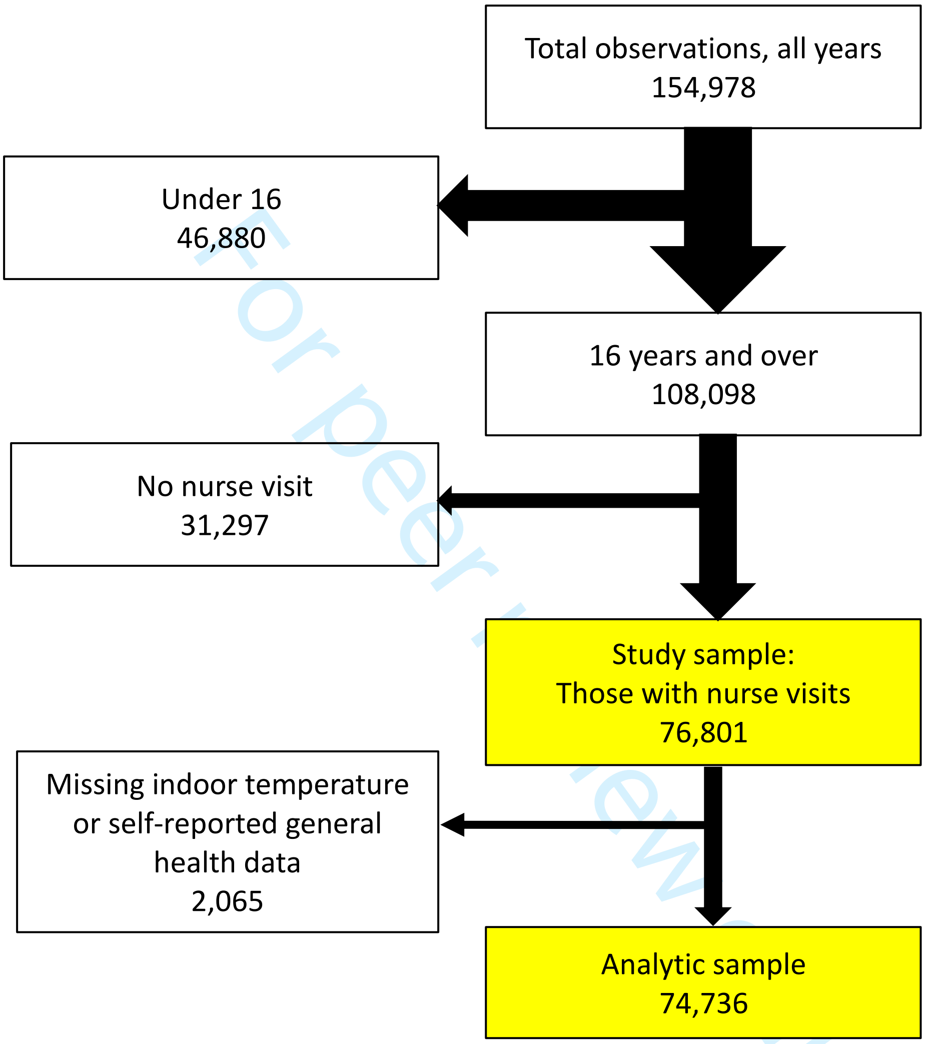

For peer review only

STROBE Statement—Checklist of items that should be included in reports of ***cross-sectional studies***

	Item No	Recommendation	This manuscript	Line no.[1]
**Title and abstract**	1	(*a*) Indicate the study's design with a commonly used term in the title or the abstract	The word 'cross-sectional' in the title; 'cross-sectional study' & 'annual … survey' in the abstract	2; 48; 53-54
		(*b*) Provide in the abstract an informative and balanced summary of what was done and what was found	Yes	52-65
**Introduction**				
Background / rationale	2	Explain the scientific background and rationale for the investigation being reported	Yes	79-128
Objectives	3	State specific objectives, including any prespecified hypotheses	Yes	129-134
**Methods**				
Study design	4	Present key elements of study design early in the paper	Yes	138-151
Setting	5	Describe the setting, locations, and relevant dates, including periods of recruitment, exposure, follow-up, and data collection	Yes	138-151; 186-238
Participants	6	(*a*) Give the eligibility criteria, and the sources and methods of selection of participants	Yes	153-178
Variables	7	Clearly define all outcomes, exposures, predictors, potential confounders, and effect modifiers. Give diagnostic criteria, if applicable	Yes	186-238
Data sources / measurement	8*	For each variable of interest, give sources of data and details of methods of assessment (measurement). Describe comparability of assessment methods if there is more than one group	Yes	186-238
Bias	9	Describe any efforts to address potential sources of bias	Yes	278-284; 387-390
Study size	10	Explain how the study size was arrived at	Yes	134-140; 147-150
Quantitative variables	11	Explain how quantitative variables were handled in the analyses. If applicable, describe which groupings were chosen and why	Yes	187-223;
Statistical methods	12	(*a*) Describe all statistical methods, including those used to control for confounding	Yes	245-284
		(*b*) Describe any methods used to examine subgroups and interactions	Yes	270-277
		(*c*) Explain how missing data were addressed	Yes	245-250
		(*d*) If applicable, describe analytical methods taking account of sampling strategy	Yes	278-284
		(*e*) Describe any sensitivity analyses	Yes	272-274
**Results**				
Participants	13*	(a) Report numbers of individuals at each stage of study—eg numbers potentially eligible, examined for eligibility, confirmed eligible, included in the study, completing follow-up, and analysed	Yes	166-178; Fig S1
		(b) Give reasons for non-participation at each stage	Yes	Fig S1
		(c) Consider use of a flow diagram	Yes	Fig S1

[1] Line numbers refer to the version with tracked changes

For peer review only - http://bmjopen.bmj.com/site/about/guidelines.xhtml

Descriptive data	14*	(a) Give characteristics of study participants (eg demographic, clinical, social) and information on exposures and potential confounders	Yes	Tab S1
		(b) Indicate number of participants with missing data for each variable of interest	N/A	147-148
Outcome data	15*	Report numbers of outcome events or summary measures	N/A	
Main results	16	(*a*) Give unadjusted estimates and, if applicable, confounder-adjusted estimates and their precision (eg, 95% confidence interval). Make clear which confounders were adjusted for and why they were included	95% CIs provided for minimally-adjusted (age and outdoor temperature) & fully adjusted models	Tab 1; Tab 2; Tab 3
		(*b*) Report category boundaries when continuous variables were categorized	Yes (age-group; tertiles of temperatures)	254-255; 271-274
		(*c*) If relevant, consider translating estimates of relative risk into absolute risk for a meaningful time period	N/A	
Other analyses	17	Report other analyses done—eg analyses of subgroups and interactions, and sensitivity analyses	Tertiles of indoor & outdoor temp	337-341; 345-348
**Discussion**				
Key results	18	Summarise key results with reference to study objectives	Yes	356-361
Limitations	19	Discuss limitations of the study, taking into account sources of potential bias or imprecision. Discuss both direction and magnitude of any potential bias	Yes	76-77; 392-403
Interpretation	20	Give a cautious overall interpretation of results considering objectives, limitations, multiplicity of analyses, results from similar studies, and other relevant evidence	Yes	361-373
Generalisability	21	Discuss the generalisability (external validity) of the study results	Yes	386-391
**Other information**				
Funding	22	Give the source of funding and the role of the funders for the present study and, if applicable, for the original study on which the present article is based	Yes	479-484

*Give information separately for exposed and unexposed groups.

Note: An Explanation and Elaboration article discusses each checklist item and gives methodological background and published examples of transparent reporting. The STROBE checklist is best used in conjunction with this article (freely available on the Web sites of PLoS Medicine at http://www.plosmedicine.org/, Annals of Internal Medicine at http://www.annals.org/, and Epidemiology at http://www.epidem.com/). Information on the STROBE Initiative is available at www.strobe-statement.org.

For peer review only

BMJ Open

Associations between indoor temperature, self-rated health and socio-economic position in a cross-sectional study of adults in England

Journal:	*BMJ Open*
Manuscript ID	bmjopen-2020-038500.R2
Article Type:	Original research
Date Submitted by the Author:	22-Dec-2020
Complete List of Authors:	Sutton-Klein, Joanna; UCL, Epidemiology and public health<br>Moody, Alison; UCL, Epidemiology and public health<br>Hamilton, Ian; UCL, UCL Energy Institute<br>Mindell, Jennifer; UCL, Epidemiology & Public Health
**Primary Subject Heading**:	Public health
Secondary Subject Heading:	Epidemiology, Health policy
Keywords:	EPIDEMIOLOGY, PUBLIC HEALTH, SOCIAL MEDICINE

SCHOLARONE™
Manuscripts

I, the Submitting Author has the right to grant and does grant on behalf of all authors of the Work (as defined in the below author licence), an exclusive licence and/or a non-exclusive licence for contributions from authors who are: i) UK Crown employees; ii) where BMJ has agreed a CC-BY licence shall apply, and/or iii) in accordance with the terms applicable for US Federal Government officers or employees acting as part of their official duties; on a worldwide, perpetual, irrevocable, royalty-free basis to BMJ Publishing Group Ltd ("BMJ") its licensees and where the relevant Journal is co-owned by BMJ to the co-owners of the Journal, to publish the Work in this journal and any other BMJ products and to exploit all rights, as set out in our licence.

The Submitting Author accepts and understands that any supply made under these terms is made by BMJ to the Submitting Author unless you are acting as an employee on behalf of your employer or a postgraduate student of an affiliated institution which is paying any applicable article publishing charge ("APC") for Open Access articles. Where the Submitting Author wishes to make the Work available on an Open Access basis (and intends to pay the relevant APC), the terms of reuse of such Open Access shall be governed by a Creative Commons licence – details of these licences and which Creative Commons licence will apply to this Work are set out in our licence referred to above.

Other than as permitted in any relevant BMJ Author's Self Archiving Policies, I confirm this Work has not been accepted for publication elsewhere, is not being considered for publication elsewhere and does not duplicate material already published. I confirm all authors consent to publication of this Work and authorise the granting of this licence.

For peer review only

Associations between indoor temperature, self-rated health and socio-economic position in a cross-sectional study of adults in England

Authors:

Dr Joanna Sutton-Klein, MB ChB, MSc, Health and Social Surveys Research Group, Research Dept of Epidemiology and Public Health, UCL, 1-19 Torrington Place, London WC1E 6BT, UK. ORCID 0000-0002-7784-0986

Alison Moody, MSc, Health and Social Surveys Research Group, Research Dept of Epidemiology and Public Health, UCL, 1-19 Torrington Place, London WC1E 6BT, UK. ORCID 0000-0002-8199-4830

Dr Ian Hamilton, PhD, UCL Energy Institute, Bartlett Faculty of the Built Environment, UCL, 14 Upper Woburn Place, London, WC1H 0NN, UK. ORCID 0000-0003-2582-2361

Prof Jennifer S. Mindell, PhD, Health and Social Surveys Research Group, Research Dept of Epidemiology and Public Health, UCL, 1-19 Torrington Place, London WC1E 6BT, UK. ORCID 0000-0002-7604-6131

Corresponding author:

Dr Ian Hamilton, UCL Energy Institute, Bartlett Faculty of the Built Environment, UCL, Central House, 14 Upper Woburn Place, London, WC1H 0NN

Email: i.hamilton@ucl.ac.uk

Telephone: +44-20-3108-5982

Research report manuscript for *BMJ Open*

Word count: 3,874

Tables: 4 plus one supplementary table

Figures: 2 plus one supplementary figure

References: 58

Subject areas: Public health; Health policy

Journal keywords: Health status, Socio-economic inequalities, Temperature

Funding

AM & JM are funded by NHS Digital to work on the annual HSE. IH is funded by EPSRC [EP/R035288/1] to work on building energy efficiency. NHS Digital and EPSRC played no part in the planning, conduct, or interpretation of these analyses; the drafting of the manuscript; nor the decision to publish.

For peer review only

Abstract

Objective

Excess winter deaths (EWDs) are a major public health concern in England and Wales, with an average of 20,000 deaths per year since 2010. Feeling cold at home during winter is associated with reporting poor general health; cold and damp homes have greater prevalence in lower socioeconomic groups. Overheating in the summer also has adverse health consequences. This study evaluates the association between indoor temperature and general health and the extent to which this is affected by socioeconomic and household factors.

Design

Cross-sectional study.

Setting

England.

Participants

Secondary data of 74,736 individuals living in England that took part in the Health Survey for England (HSE) between 2003 and 2014. The HSE is an annual household survey which uses multilevel stratification to select a new, nationally-representative sample each year. The study sample comprised adults who had a nurse visit; the analytical sample was adults who had observations for indoor temperature and self-rated health.

Results

Using both logistic and linear regression models to examine indoor temperature and health status, adjusting for socio-economic and housing factors, the study found an association between poor health and higher indoor temperatures. Each one degree increase in indoor temperature was associated with a 1·7% (95% Confidence Interval (CI) 0·7%-2·6%) increase in the odds of poor health. After adjusting for income, education, employment type, household size and home ownership, the odds ratio (OR) of poor health for each degree temperature rise increased by 19%, to 1·02 (95%CI 1·01-1·03 P<0·01).

Conclusion

People with worse self-reported health had higher indoor temperatures after adjusting for household factors. People with worse health may have chosen to maintain warmer environments or been advised to. However, other latent factors, such as housing type and energy performance could have an effect.

Article summary: Strengths and limitations of this study

- This study is based on data from 74,736 individuals, randomly sampled from the general population to be nationally-representative of the population in England.
- Temperature measurements were taken using a standard protocol across the 10 years.
- Analyses used individual-level data, not ecological comparisons.
- Indoor temperatures were measured only once per participant.
- Data are cross-sectional, so causality cannot be inferred.

For peer review only - http://bmjopen.bmj.com/site/about/guidelines.xhtml

For peer review only

Introduction

Short-term exposure to extreme hot or cold temperatures is known to be acutely harmful. For example, Analitis *et al.* compared the short-term effects of cold on mortality in 15 European cities, finding that a 1°C decrease in outdoor temperature was associated with a 1·35% increase in the daily number of total natural deaths.[1] A recent systematic review has shown the adverse health consequences of high indoor temperatures, above 26°C.[2] However, the long-term effects of exposure to different temperatures are not well understood. Much of the evidence on long-term exposure to more minor deviations in ambient temperature is overwhelmingly drawn from ecological studies.[3–5]

There were 49,410 excess winter deaths (EWDs) in England and Wales over the 2017-18 winter.[6] Although EWDs were significantly lower in 2018-19 (23,200, provisional figure),[6] the large inequalities in EWD index between European countries suggest a major public health problem.[7] Paradoxically, while several studies have found that cooler ambient temperatures are associated with higher mortality on a day-by-day analysis, European countries with colder winters were found to have lower excess winter deaths.[8] Studies have suggested that it is the variation in housing quality and the resulting indoor temperature differences which may instead be the drivers of these differences in health outcomes.[9,10]

Few studies have directly addressed the association between indoor temperature and health in individuals, but a number have studied known correlates of indoor temperature such as damp, insulation, or perceived thermal comfort.[11,12] These studies overwhelmingly found that proxies for cool indoor temperature were associated with worse health.

Several studies have compared the health of individuals before and after housing interventions. Upgraded insulation in New Zealand was associated with increased indoor temperature, and lower levels of poor general health (odds ratio, (OR) 0·50, 95% confidence interval (CI) 0·38-0·68).[13,14] Furthermore, a large pan-European study found that perceived coldness at home was strongly associated with poor general health, with an OR of 2.6 (95% CI 2.1-3.1),[15] a result which was replicated in a study of English adults.[16] A limitation of using perceived thermal comfort as a proxy of indoor temperature is that its subjectivity makes it vulnerable to reverse causation.

Analysis of the 1991 survey of English houses found that the strongest predictors of low indoor temperatures were age of property, absence of central heating, cost of heating, small household size (i.e. fewer occupants), and low income.[17] A 2011 survey of English houses found similar importance of predictors of indoor temperature; that older properties were not the coldest; but that low energy performance ratings and low income households had the lowest temperatures.[18]

A systematic review by Public Health England concluded that living in cold temperatures (below 18°C) may increase the risk of adverse health consequences, particularly for premature mortality in vulnerable populations.[19] However, recommendations for minimum indoor temperature are based on limited evidence.[15] Research has shown that few households are keeping the recommended indoor temperatures (i.e. ≥18°C), particularly vulnerable households.[20] Recently, increasing poverty was associated with higher risk of poor housing within the UK.[21,22] Analysis of Census longitudinal study data has shown a rise in the proportion of homes with central heating from 83% in 1991, to 93% in 2001 and 98% in 2011.[23] The English Housing Survey in 2016 found that prevalence of cavity or solid wall insulation, of double glazing, and of central heating had increased in England to

For peer review only - http://bmjopen.bmj.com/site/about/guidelines.xhtml

49%, 83%, and 92% of households respectively.[24] A study on government interventions found that the installation of heating and insulation was effective at increasing indoor temperatures.[25] There are also deaths in England due to excessive temperatures during heatwaves,[26] so both high and low indoor temperatures warrant studying in relation to associations with health, although cold is the primary focus because that is the dominant exposure in England.[27]

The aim of our study was to investigate the relationship between indoor temperature and general health, and to examine the extent to which socioeconomic and housing variables explain any association found. We hypothesised that: socioeconomic and housing factors would be associated with indoor temperature; low indoor temperature would be associated with worse general health; and the association between temperature and general health would be stronger with decreasing socioeconomic status.

Methods

Study design

This study is based on the Health Survey for England (HSE), an annual, cross-sectional, household, health examination survey which has run since 1991. A new, nationally-representative sample of the free-living general population is selected each year, using a stratified, two-stage selection process. The sampling frame is the small addresses Postal Address File. The survey varied in size each year from around 9,000 to 16,000 households in the study sample. Data were collected first by an interviewer, who also measured height and weight; a nurse visit followed for participants who agreed. The nurse asked further questions and took biophysical measurements, including blood pressure. More details are reported elsewhere,[28,29] including the Methods report for the annual report published each year (e.g.[30]).

For this survey, we used data from each year HSE 2003 to HSE 2014 excluding 2004, when the core general population sample did not have a nurse visit. These years were selected because identical variables have been coded consistently from 2003 onwards; 2014 was the most recent dataset available in the UK Data Archive at the time the analyses commenced.

Participants

The study sample was adults aged 16 years and over who took part in the nurse visit component of the HSE in 2003 or 2005 to 2014 . The study sample comprised adults who had a nurse visit; the analytical sample was adults who had observations for indoor temperature and self-rated health (Supplementary Figure S1). The analytic sample (n=74,736) was compared with those excluded from the study sample (n=2,065, Table 1, Supplementary Table S1). There were no statistically significant differences between the analytic sample and those excluded regarding mean monthly outdoor temperatures by region (data not shown). However, all categorical variables, except for respiratory disease, had significant differences between the analytic sample and the excluded participants. The analytic sample comprised 55% women, while 74% of the excluded participants were women (P<0·01). The excluded survey participants had a younger age profile than the analytic sample; were less likely to be in the top two income quintiles; were more likely to live in social housing; but were more likely to have degree level qualifications. The analytic

For peer review only - http://bmjopen.bmj.com/site/about/guidelines.xhtml

sample were more likely to own their homes, and to have a longstanding illness or cardiovascular condition.

Research ethics approval

Research ethics approval was obtained prior to each year's survey from the relevant research ethics committees. Participants gave informed consent at each stage of the survey. Ethical approval was not required for this specific study, which was conducted on non-identifiable, archived data held by the UK Data Service for widespread research use.

Data

General health

General health was a self-assessed variable, referred to as self-rated health. It was recorded at the interview stage, prior to the nurse visit. The participants selected one of the five answer options *"Very good, good, fair, bad or very bad?"* to the question: *"How is your health in general?"* The wording of this question, which has been used throughout the Health Survey for England series, is identical to that recommended by the WHO in its 1996 report on harmonisation of instruments in health interview surveys[31]; the ONS (the Office for National Statistics) in 2008[32]; and the UK Government Statistical Service harmonisation.[32] For this analysis, general health was dichotomised into 'good', which encompassed the categories very good and good, and 'poor', which included fair, bad and very bad. Fair was placed in the 'poor' category rather than the 'good' category as the distribution of general health is highly skewed: 74% of participants reported good or very good general health.

Poor health

The other self-reported health data used in these analyses included the presence of limiting or non-limiting longstanding illness; a respiratory condition; or a cardiovascular condition. Participants were first asked if they had any longstanding illness or condition. Those who responded positively were then asked if it limited their daily activities, and asked what the condition(s) were, to a maximum of six. These were then coded into ICD-10 chapters, from which responses from participants volunteering a respiratory condition (chapter J) and/or a cardiovascular condition (chapter I) were identified.

Demographic data

Gender and age were collected at the interview.

Socio-economic data

Three variables were used for socio-economic position. Education level was categorised as degree or equivalent, qualification below degree, or no qualification. Equivalised household income, which adjusts for the numbers of adults and of children in the household, was divided into quintiles. The National Statistics-Socio-Economic Classification of occupation in five categories (NS-SEC5)[33] was derived from detailed information about employment status, occupation, size of organisation, and managerial responsibility. The categories were managerial & professional, intermediate, small employers, lower supervisory & technical, and semi-routine.

For peer review only - http://bmjopen.bmj.com/site/about/guidelines.xhtml

Housing

Housing information comprised the number of occupants in the household, and housing tenure. The latter was categorised as owner-occupied (e.g. owned outright or with a mortgage), rented privately, or socially rented (i.e. from a housing association or local government).

Temperature

Indoor air temperature was measured by the nurse, using a digital thermometer with a probe, prior to measuring blood pressure. The thermometer was placed on a surface near the blood pressure equipment, away from a radiator and out of direct sunlight. Nurses were recommended that the probe hang over the edge of the table.

Mean monthly outdoor temperature data by region were obtained from the Met Office website. The data are publicly available. The Met Office is a governmental agency in the UK which provides weather forecasts and warnings. The data were measured according to their standard protocol at weather stations 40 kilometres apart across the country, every hour.

Data sharing

The pseudonymised HSE data from each survey are deposited in the UK Data Archive and are available to be downloaded from the UK Data Service at: beta.ukdataservice.ac.uk/datacatalogue/series/series?id=2000021.

Statistical analysis

For the variables NS-SEC5, income and tenure, answers in the ‘other’ category, or categories outside of the main analytical categories (e.g. unemployed, student, or homemaker for NS-SEC) were grouped into an additional category of ‘other or missing’ and included in the models. This was because there were many participants in those categories and excluding them would have substantially decreased the sample size. The data were combined into a single dataset.

The analysis was run in Stata v15 (StataCorp, 2017). Models were built to test each hypothesis. Each model used the same analytical sample. The models were built using maximum likelihood estimation, and the best models were selected using the likelihood ratio test. Age was grouped for analysis as 16-24 then 10y groups to 75+, to avoid prior assumptions of the shape of the relationship.

Associations between socioeconomic and housing variables and indoor temperature were explored in two ways. Logistic regression models were created with indoor temperature <18℃ as the outcome variable (compared with indoor temperature of 18℃ or above) as well as linear regression models with indoor temperature as a continuous variable. For both sets of models, socioeconomic and housing variables were included as explanatory variables. Missing data among the covariates were included in the regression as an additional category.

To test the association of indoor temperature with general health, a similar set of logistic regression models were created but with indoor temperature as the explanatory variable, and general self-reported health as the outcome variable, again adjusting for demographic, housing, and socio-economic variables. The models were run for the whole sample, then

For peer review only - http://bmjopen.bmj.com/site/about/guidelines.xhtml

were then repeated stratified by tertile of outdoor temperature to assess if the association differed across the range of outdoor temperatures. As a sensitivity analysis, model 9 (fully adjusted) was repeated stratified by tertile of indoor temperature to assess if the association differed across the range of indoor temperatures.

The unadjusted coefficients of the variables are reported, as well as the coefficients after adjusting for other socioeconomic and housing variables. Collinearity was assessed by looking for reversal of the sign of the coefficients, and the variance inflation factor.

The analysis was weighted using the survey-provided weights, which account for probabilities of selection (due to the sample design), and response rate differences by age, sex, and region. This ensured a weighted sample which matched the ONS-mid-year population estimates by age and sex for each survey year.[30] The multi-stage survey design was accounted for using the ‘svy’ commands in STATA, using the survey’s primary sampling units, and using government office region as the strata variable, since this was a component of stratification that was consistent across the survey years.

Results

The analytical sample consisted of 74,736 participants, 97·3% of the study sample. Their characteristics are shown in Table 1 and Supplementary Table S1. The indoor temperature ranged from 7·5°C to 36·8°C, with a mean of 20·7°C (SD 2·3). The outdoor temperature ranged from -1·2ºC to 20·5ºC, with a mean of 10·1ºC (SD 4·6). For both indoor and outdoor temperature, most of the variation was attributable to month. 10% of households had indoor temperatures below the UK government’s recommended minimum of 18°C.

<Insert Table 1 around here>

Table 1. Distribution of categorical variables by complete and incomplete cases within the study sample, and P-values for Chi-squared tests (unweighted)

Variable		Incomplete (n=2,065)		Complete (n=74,736)		χ^2 P-value
		N	%	N	%	
Sex	Men	533	26	33,526	45	<0.01
	Women	1,532	74	41,210	55	
Age group	16-24	373	18	6,539	9	<0.01
	25-34	639	31	9,586	13	
	35-44	375	18	13,145	18	
	45-54	179	9	12,929	17	
	55-64	161	8	12,753	17	
	65-74	162	8	11,315	15	
	75+	176	9	8,412	11	
NS-SEC5	Managerial/professional	557	27	25,297	34	<0.01
	Intermediate Occupation	234	11	10,354	14	
	Small employers	9	0	6,514	9	
	Lower supervisory	96	5	6,087	8	

For peer review only - http://bmjopen.bmj.com/site/about/guidelines.xhtml

	Semi-routine	511	25	23,314	31	
	Missing	*658*	*32*	*3,170*	*4*	
Education	Degree level or higher	476	24	15,400	21	<0.01
	Qualification below degree	992	50	40,233	54	
	No qualification	515	26	19,103	26	
Income quintile	Lowest quintile	420	20	10,861	15	<0.01
	2nd lowest	302	15	12,115	16	
	3rd lowest	260	13	13,190	18	
	4th lowest	265	13	13,537	18	
	Highest quintile	321	16	13,291	18	
	Missing	*497*	*24*	*11,742*	*16*	
Tenure	Owner-occupied	1,076	52	55,159	74	<0.01
	Privately rented	283	14	6,476	9	
	Socially rented	492	24	11,347	15	
	Missing	*214*	*10*	*1,174*	*2*	
Household size	1	284	14	13,677	18	<0.01
	2	712	35	30,117	40	
	3	540	26	12,585	17	
	4	323	16	12,173	16	
	5+	206	10	6,184	8	
General health	Fair	1,555	76	55,235	74	0.06
	Poor	498	24	19,501	26	
	Missing	*12*	*1*	*0*	*0*	
Longstanding illness	No	1,269	62	39,246	53	<0.01
	Yes	781	38	35,490	48	
	Missing	*15*	*1*	*0*	*0*	
Heart condition	No	1,838	90	63,905	86	<0.01
	Yes	212	10	10,831	14	
	Missing	*15*	*1*	*0*	*0*	
Respiratory condition	No	1,869	91	68,051	91	0.86
	Yes	181	9	6,685	9	
	Missing	*15*	*1*	*0*	*0*	
Total		2,065	3	74,736	97	

Socioeconomic and housing variables and indoor temperature

Households with more than one occupant had a lower odds of an indoor temperature <18°C (Table 2) and higher mean indoor temperatures (Tables 3 and S1) than single person households, as did households with higher equivalised household incomes compared with those with lower incomes. The adjusted OR (Table 2) and β-coefficient (Table 3) for both household size and income showed a gradient across all categories. Higher educational attainment, and private sector rented houses (compared with owner-occupied housing) were associated with lower mean indoor temperatures (Table 3) and a higher odds of the indoor temperature being <18°C (Table 2). NS-SEC5 was variably associated with low temperature:

For peer review only

compared with those in managerial and professional occupations, we found reduced adjusted OR among those in intermediate occupations but higher odds among small employers, and with mean indoor temperature (positive association in lower supervisory and technical households but inverse association among small employers) in the fully adjusted models.

<Insert Table 2 around here>

<Insert Table 3 around here>

For peer review only

For peer review only - http://bmjopen.bmj.com/site/about/guidelines.xhtml

Table 2 - Logistic regression models of associations of socioeconomic and housing variables with low indoor temperature (<18°C)

	Minimally adjusted[a]				Fully adjusted[b]			
	OR	95% CI		P	aOR	95% CI		P
**Household size**								
1	1				1			
2	0·58	0·54	0·63	<0·001	0·59	0·54	0·63	<0·001
3	0·46	0·41	0·51	<0·001	0·46	0·41	0·51	<0·001
4	0·38	0·34	0·43	<0·001	0·38	0·34	0·43	<0·001
5+	0·37	0·32	0·44	<0·001	0·36	0·31	0·43	<0·001
**Tenure**								
Owner-occupied	1				1			
Privately rented	1·49	1·33	1·68	<0·001	1·27	1·12	1·43	<0·001
Socially rented	1·10	1·00	1·20	0·056	0·94	0·85	1·05	0·264
Other or missing	1.39	1.15	1.68	0.001	1.18	0.98	1.43	0.086
**NS-SEC5**								
Managerial & professional	1				1			
Intermediate	0·90	0·83	0·98	0·015	0·92	0·84	1·00	0·047
Small employers	1·17	1·06	1·29	0·003	1·20	1·09	1·33	<0·001
Lower supervisory & technical	0·91	0·81	1·01	0·083	0·93	0·83	1·05	0·236
Semi-routine	1·00	0·94	1·08	0·901	1·02	0·94	1·11	0·585
Other or missing	1.00	0.85	1.18	0.980	1.12	0.95	1.32	0.165
**Education**								
Degree	1				1			
Qualification below degree	0·91	0·85	0·98	0·017	0·92	0·85	1·00	0·045
No qualification	0·90	0·82	0·99	0·029	0·84	0·75	0·93	0·001
**Equivalised household income quintile**								
Lowest quintile	1				1			
2nd quintile	0·87	0·78	0·98	0·024	0·91	0·81	1·03	0·126
3rd quintile	0·86	0·77	0·96	0·010	0·88	0·78	0·99	0·030
4th quintile	0·84	0·75	0·95	0·004	0·83	0·73	0·94	0·003
Highest quintile	0·86	0·77	0·97	0·013	0·77	0·67	0·88	<0·001
Other or missing	0.87	0.77	0.99	0.035	0.90	0.79	1.03	0.112

[a] Minimally adjusted models: adjusted for outdoor temperature and age group.

[b] Fully adjusted models: mutually adjusted for all socioeconomic and housing variables in this table.

For peer review only

Table 3 - Linear regression models with socioeconomic and housing variables as explanatory variables for indoor temperature

	Minimally adjusted[a]				Fully adjusted[b]			
	β	95% CI		P	β	95% CI		P
**Household size**								
1	Ref							
2	0·41	0·35	0·46	<0·001	0·43	0·38	0·49	<0·001
3	0·63	0·56	0·71	<0·001	0·65	0·58	0·73	<0·001
4	0·74	0·66	0·82	<0·001	0·76	0·68	0·85	<0·001
5+	0·79	0·68	0·90	<0·001	0·81	0·70	0·93	<0·001
**Tenure**								
Owner-occupied	Ref							
Privately rented	-0·23	-0·32	-0·14	<0·001	-0·11	-0·20	-0·02	0·017
Socially rented	0·13	0·06	0·19	<0·001	0·21	0·14	0·28	<0·001
Other or missing	-0.12	-.028	0.04	0.146	-0.01	-0.17	0.15	0.918
**NS-SEC5**								
Managerial and professional occupations	Ref							
Intermediate	0·08	0·03	0·13	0·003	0·03	-0·03	0·08	0·298
Small employers	-0·05	-0·12	0·02	0·177	-0·11	-0·18	-0·04	0·001
Lower supervisory and technical	0·18	0·11	0·24	<0·001	0·09	0·02	0·16	0·010
Semi-routine	0·06	0·01	0·11	0·020	-0·04	-0·09	0·01	0·128
Other or missing	0.11	0.01	0.22	0.040	-0.06	-0.17	0.04	0.250
**Education**								
Degree	Ref							
Qualification below degree	0·16	0·11	0·21	<0·001	0·14	0·08	0·19	<0·001
No qualification	0·22	0·16	0·29	<0·001	0·22	0·15	0·29	<0·001
**Income quintile**								
Lowest quintile	Ref							
2nd quintile	0·03	-0·05	0·11	0·468	0·04	-0·05	0·12	0·402
3rd quintile	0·02	-0·06	0·10	0·631	0·07	-0·01	0·16	0·081
4th quintile	0·01	-0·07	0·09	0·843	0·11	0·02	0·19	0·016
Highest quintile	-0·04	-0·12	0·05	0·402	0·15	0·06	0·24	0·001
Other or missing	0.05	-0.04	0.14	0.315	0.08	-0.01	0.17	0.091

[a] Minimally adjusted models: adjusted for outdoor temperature and age group.

[b] Fully adjusted models: mutually adjusted for all socioeconomic and housing variables in this table.

For peer review only

For peer review only - http://bmjopen.bmj.com/site/about/guidelines.xhtml

Indoor temperature and health

Each one degree increase in indoor temperature was associated with a 1.4% (95% CI 0.7-2.6) increase in odds of poor health, after adjusting for age and gender (see Figure 1). After adjusting for socioeconomic and housing factors, each degree increase in temperature is associated with a 1.7% (95% CI 0.7-2.6) increase in odds of poor health.

<Insert Figure 1 around here>

A series of regression models were run to examine the impact on the odds of reporting poor health associated with indoor temperature of demographic, housing, and socio-economic factors (Table 4). Adjusting for household size increased the odds ratio most, with small changes for household size and tenure or for equivalised household income . Adjusting for tenure, NS-SEC5, or all three socio-economic factors reduced the odds ratio a little. The linear association between indoor temperature and reporting poor health remained significant in each model; the final model, including all these covariates, also increased the odds ratio compared with the base model.

When stratified by indoor temperature range, and adjusting for socioeconomic and housing factors, the odds ratios for each indoor temperature tertile did not differ significantly from one another (sensitivity analysis, data not shown). With the truncated range of temperatures, the relationship between indoor temperature and poor health was no longer significant within the upper two tertiles. This may be due to truncation in the distribution of indoor temperature, meaning that only the lowest tertile included the low temperatures associated with poor health.

When regression models were repeated stratified for tertile of outdoor temperature, we found the ORs of indoor temperature on poor health were stronger for the coldest tertile (i.e. <7˚C). For example, the adjusted Odds Ratios for Models 7 and 9, which included adjustment for housing variables, were 1.04 (1.02-1.05) in the lowest tertile but were not significantly different from 1.0 for the middle and highest tertile of outdoor temperature (Table 4). This may be because the lower the outdoor temperature, the more likely it is that the occupant will choose to turn on their heating and therefore choose what temperature their home is at, which will depend on the characteristics of both the home and the residents. On warmer days, occupants are less likely to turn on their heating, and therefore the indoor temperature will be determined more by the outdoor temperature than the characteristics of the occupant, and as such will be less associated with any characteristics of the occupant.

<Insert Table 4 around here>

For peer review only - http://bmjopen.bmj.com/site/about/guidelines.xhtml

Table 4 - Logistic regression models showing odds ratios of poor health for indoor temperature, stratified by tertile of outdoor temperature

	All				Lowest tertile of outdoor temperature (<7˚C)				Middle tertile of outdoor temperature (7-12.8˚C)				Highest tertile of outdoor temperature (≥12.9˚C)			
**Indoor temperature[a,b]**	**aOR**	**95% CI**		**P**	**aOR**	**95% CI**		**P**	**aOR**	**95% CI**		**P**	**aOR**	**95% CI**		**P**
Model 1: adjusted for age and gender	1·01	1·00	1·02	0·003	1.02	1.01	1.04	0.004	1.01	1.00	1.03	0.122	1.02	1.00	1.03	0.069
Model 2: Model 1 + Household size	1·02	1·01	1·03	<0·001	1.04	1.02	1.05	<0·001	1.02	1.00	1.04	0.018	1.02	1.00	1.03	0.032
Model 3: Model 1 + Tenure	1·01	1·00	1·02	0·014	1.03	1.01	1.05	0.001	1.01	0.99	1.03	0.275	1.00	0.99	1.02	0.614
Model 4: Model 1 + NS-SEC5	1·01	1·00	1·02	<0·005	1.02	1.01	1.04	0.007	1.02	1.00	1.03	0.091	1.01	1.00	1.03	0.163
Model 5: Model 1 + Income	1·02	1·01	1·03	<0·001	1.03	1.01	1.05	0.001	1.01	1.00	1.03	0.138	1.01	1.00	1.03	0.117
Model 6: Model 1 + Education	1·01	1·00	1·02	0·034	1.02	1.00	1.03	0.029	1.01	0.99	1.03	0.276	1.01	0.99	1.03	0.208
Model 7: Model 1 + housing variables	1·02	1·01	1·02	0·002	1.04	1.02	1.05	<0·001	1.01	1.00	1.03	0.132	1.01	0.99	1.02	0.470
Model 8, Model 1 + socioeconomic variables	1·01	1·00	1·02	0·007	1.02	1.01	1.04	0.004	1.01	0.99	1.03	0.191	1.01	0.99	1.03	0.310
Model 9: Model 1 + housing and socioeconomic variables	1·02	1·01	1·03	<0·001	1.04	1.02	1.05	<0·001	1.02	1.00	1.04	0.086	1.01	0.99	1.02	0.465

[a] Indoor temperature was linear in these models.

[b] Models are listed in order of increasing likelihood. All models adjusted for age group and gender; Model 7 as Model 1 plus household size and tenure; Model 8 as Model 1 plus NS-SEC5, equivalised income quintiles, and education; Model 9 included all the variables.

For peer review only

Discussion

The results showed an association between higher indoor temperatures and increased odds of poor self-rated health. This has also been shown recently in a small number of individuals in Cornwall, UK: participants with poorer health maintained their homes at higher temperatures.[34] However, the relationship between socioeconomic and housing factors with indoor temperature was complicated, with different measures having different directions of association with indoor temperature (Figure 2). The most intuitive association is that those with lower incomes tended to have lower indoor temperatures, possibly because they cannot afford to pay for gas or electricity to heat their homes. This may explain why the association between indoor temperature and poor health was strongest when the outdoor temperature was lower. Conversely, other markers of lower social class, including lower NS-SEC5 and lower education, were associated with warmer indoor temperatures. These differing associations might be best explained by the finding that privately rented accommodation was cooler than homes that were socially rented. This is a reflection that the ability to raise indoor temperature is determined by both the financial ability to pay for fuel and the insulation of the home. Socially rented accommodation tends to be better insulated than privately rented accommodation both because of the housing quality and space standards required on social housing[35] and because social landlords such as housing associations and local councils may have more incentive to provide decent quality accommodation as a longterm asset. A small study in Japan, using perceived thermal comfort not measured temperature, found that people who reported living in cold homes had a higher risk of frailty - but only among those who were dissatisfied with their economic situation.[36]

For peer review only

It is not clear from this research why temperatures are higher among households with worse self-rated health and whether this is due to personal preferences and behaviours; advice from medical or other professionals to maintain a warmer home; the difference in the type of building they occupy (i.e. dwellings that are typically warmer such as smaller houses or flats, and social housing rather than privately rented); or some combination of these. Figure 2 shows a conceptual framework for the way in which these variables affect the associations.

<Figure 2 around here>

The strengths of this study are the large number of participants, who were nationally-representative of the non-institutionalised population living in England. The temperature measurements were taken using a standard protocol across the time period but were not the focus of the surveys, so the likelihood of differential response rates systematically associated with indoor temperature is very small. Unlike the study by Armstrong and colleagues, we report data based on individuals, rather than ecological analyses.[37]

Limitations include the significant differences in many characteristics between the analytic sample, who had indoor measurements, and those excluded because there were no measurements. The latter comprises those who were ineligible for blood pressure measurement (for example being pregnant) or declined the measurement. However, fewer than 3% of the study sample were excluded; the analytic sample is therefore almost identical to the study sample overall. In addition, there was some missing data among the analytic sample (Table 1). Income is the variable with the highest level of missingness. It is likely that this group contains disproportionate numbers of households both of high and of low income.

For peer review only - http://bmjopen.bmj.com/site/about/guidelines.xhtml

Tables 2 and 3 show that this group have a similar odds of low temperature as the 2nd lowest quintile, and a similar coefficient for linear temperature as the 3rd quintile in the fully adjusted models.

The third main limitation was the measurement of indoor temperature at a single time point for each participant in whichever room they were 'hosting' the survey nurse, generally in the living room or kitchen. Huebner and colleagues have shown four different profiles in living room temperature variability across the day, varying from bimodal (morning and evening peaks), steadily increasing (shallow and steep) to flat with little variation.[38] Using the single time point temperature reading in the analysis requires an assumption that the snapshot temperature reading for each participant reflects an average temperature for the home over a significant period of time.

Hajat and colleague have shown that 40% of cold-related deaths occur outside the December-March period used for calculating 'excess winter deaths'.[27] For that reason, and given seasonal and temperature differences across the regions of England, we used tertile of outdoor temperature rather than season of other groupings of months. We showed a strong relationship at low outdoor temperatures but little if any effect at high outdoor temperatures. Impacts of heatwaves are generally acute, so it is less likely that we would have found a strong relationship at higher outdoor temperatures.

While the subjectivity of self-rated health makes it vulnerable to information bias, it has been shown to be highly predictive of future mortality and health outcomes, and is used frequently across the scientific literature.[39,40] Self-rated health is strongly associated with total mortality [41–45] and with some specific causes of death[46,47]but not with external causes.[46] It also predicts functional ability.[48] Self-rated health is explained more by measures of mental and physical health than by demographic or socio-economic factors.[49]

Apart from the well-known association between temperature and blood pressure,[50–53] there is less evidence pointing at more specific health outcomes related to temperature. Thus, as this is the first study of its kind, the broadness of general health as the outcome was a strength rather than a weakness. The differences between the entire sample and those with complete data on self-rated health and temperature was primarily due to non-response to the nurse visit among interviewees. This was dealt with by use of non-response weighting.[29]

While guidelines on healthy indoor temperatures have been issued by the World Health Organisation (WHO) and the UK Public Health England, they appear arbitrary and the WHO itself describe its own temperature recommendations as *'[scientifically] weak and not well-known to the public or policy makers'*.[54–56] Since 2014, the recommendation from the UK government is for a minimum of 18°C in all rooms, a change from the previous 21°C for living rooms.[57] The government attributed the change to a review of evidence, awareness of climate change and financial difficulties, and acceptability (*"We know that people have strong feelings about their homes and don't want to be told what to do in them")*.[56] Further research is needed to understand the temporality and mechanism of the association.

A recent study in England found no impact of Winter Fuel Payment eligibility on indoor temperature,[58] but no study has examined at individual level the impacts of energy efficiency retrofits on indoor temperature, residents' heating-relate behaviours, or health. When linked data are available, we intend to examine the associations of indoor temperature

For peer review only - http://bmjopen.bmj.com/site/about/guidelines.xhtml

For peer review only

with subsequent hospital admissions and mortality. This will be important for assessing and making health-related policy recommendations for indoor temperature. Future research in this area could take advantage of internet-enabled ‘smart’ thermostats.

For peer review only

For peer review only - http://bmjopen.bmj.com/site/about/guidelines.xhtml

Figure captions

Figure 1 - Predicted prevalence of poor health for each degree increase in indoor temperature

Footnote for Figure 1:

Prevalence of poor health predicted by the logistic regression of indoor temperature on poor health, adjusted for age and sex. OR 1.01 (95% CI 1.00-1.02), see Table 4, model 1.

Figure 2 - Conceptual framework for temperature-health association

For peer review only

For peer review only - http://bmjopen.bmj.com/site/about/guidelines.xhtml

Acknowledgements

We thank the nurses and participants in the Health Surveys for England, and our colleagues at NatCen Social Research and UCL.

Author contributions

This work was conducted as part of JS-K's MSc in Social Epidemiology. She led on conducting the literature search, designing and conducting the analyses, creating the figures, and interpreting the data. AM and JM contributed advice to the dissertation. JS-K and JM wrote the initial draft of the manuscript. IH updated the literature search and contributed to analysis and preparation of the manuscript. All authors contributed to subsequent drafts, approved the final manuscript, and are accountable for the accuracy and integrity of all aspects of the work.

Patient and Public Involvement

This study does not involve patients as the data come from a cross-section of the general population, regardless of their involvement (or not) with any health services. The public were not involved in the design of this study but the Health Survey for England itself has an Advisory Group that includes stakeholders from local government, public health, the Department of Health and Social Care, Public Health England, NHS Digital, and academe.

Age UK, Kilburn Older Voices (KOVE), the Department of Business, Energy and Industrial Strategy (BEIS), the Met Office, NatCen Social Research, and Public Health England will help with the dissemination of these findings.

Role of the funding source

NHS Digital, which funds the HSE, also funds AM & JM to work on the annual HSE. This work was also supported by the EPSRC [EP/R035288/1] to fund IH to work on home energy efficiency. NHS Digital and EPSRC played no part in the planning, conduct, or interpretation of these analyses; the drafting of the manuscript; nor the decision to publish.

Conflict of interest statement

Professor Mindell and Ms. Moody report grants from NHS Digital during the conduct of the study; Dr. Sutton-Klein has nothing to disclose. Dr. Hamilton report grants from the Engineering and Physical Sciences Research Council (EPSRC).

For peer review only

References

1 Analitis A, Katsouyanni K, Biggeri A, *et al.* Effects of Cold Weather on Mortality: Results From 15 European Cities Within the PHEWE Project. *American Journal of Epidemiology* 2008;**168**:1397–408. doi:10.1093/aje/kwn266

2 Tham S, Thompson R, Landeg O, *et al.* Indoor temperature and health: a global systematic review. *Public Health* 2019;**179**:9–17. doi:10.1016/j.puhe.2019.09.005

3 Marmot Review Team. the health impacts of cold homes and fuel poverty. London: : Friends of the Earth 2011. http://www.instituteofhealthequity.org/resources-reports/the-health-impacts-of-cold-homes-and-fuel-poverty/the-health-impacts-of-cold-homes-and-fuel-poverty.pdf (accessed 14 Jul 2020).

4 Hammel HT, Pierce JB. Regulation of Internal Body Temperature. *Annu Rev Physiol* 1968;**30**:641–710. doi:10.1146/annurev.ph.30.030168.003233

5 Mallet ML. Pathophysiology of accidental hypothermia. *QJM* 2002;**95**:775–85. doi:10.1093/qjmed/95.12.775

6 ONS. Excess Winter Mortality in England and Wales : 2018/19 (Provisional) and 2017/18 (Final). ONS 2019. https://www.ons.gov.uk/peoplepopulationandcommunity/birthsdeathsandmarriages/deaths/bulletins/excesswintermortalityinenglandandwales/2018to2019provisionaland2017to2018final

7 Liddell C, Morris C, Thomson H, *et al.* Excess winter deaths in 30 European countries 1980–2013: a critical review of methods. *J Public Health* 2015;:fdv184. doi:10.1093/pubmed/fdv184

8 Laake K, Sverre JM. Winter Excess Mortality: A Comparison between Norway and England plus Wales. *Age Ageing* 1996;**25**:343–8. doi:10.1093/ageing/25.5.343

9 Fowler T, Southgate RJ, Waite T, *et al.* Excess Winter Deaths in Europe: a multi-country descriptive analysis. *European Journal of Public Health* 2015;**25**:339–45. doi:10.1093/eurpub/cku073

10 Healy JD. Excess winter mortality in Europe: a cross country analysis identifying key risk factors. *Journal of Epidemiology & Community Health* 2003;**57**:784–9. doi:10.1136/jech.57.10.784

11 Collins KJ. Low indoor temperatures and morbidity in the elderly. *Age Ageing* 1986;**15**:212–20. doi:10.1093/ageing/15.4.212

12 Clinch JP. Housing standards and excess winter mortality. *Journal of Epidemiology & Community Health* 2000;**54**:719–20. doi:10.1136/jech.54.9.719

13 Howden-Chapman P, Matheson A, Crane J, *et al.* Effect of insulating existing houses on health inequality: cluster randomised study in the community. *BMJ* 2007;**334**:460. doi:10.1136/bmj.39070.573032.80

14 Lloyd EL, McCormack C, McKeever M, *et al.* The effect of improving the thermal quality of cold housing on blood pressure and general health: a research note. *Journal of Epidemiology & Community Health* 2008;**62**:793–7. doi:10.1136/jech.2007.067835

For peer review only

15 Ormandy D, Ezratty V. Health and thermal comfort: From WHO guidance to housing strategies. *Energy Policy* 2012;**49**:116–21. doi:10.1016/j.enpol.2011.09.003

16 Evans J. An epidemiological study of the relative importance of damp housing in relation to adult health. *Journal of Epidemiology & Community Health* 2000;**54**:677–86. doi:10.1136/jech.54.9.677

17 Wilkinson P, Landon M, Armstrong B, *et al.* Cold comfort: the social and environmental determinants of excess winter death in England, 1986-1996. York: : Joseph Rowntree Foundation 2001. https://researchonline.lshtm.ac.uk/id/eprint/17944

18 Hamilton IG, O'Sullivan A, Huebner G, *et al.* Old and cold? Findings on the determinants of indoor temperatures in English dwellings during cold conditions. *Energy and Buildings* 2017;**141**:142–57. doi:10.1016/j.enbuild.2017.02.014

19 Jevons R, Carmichael C, Crossley A, *et al.* Minimum indoor temperature threshold recommendations for English homes in winter – A systematic review. *Public Health* 2016;**136**:4–12. doi:10.1016/j.puhe.2016.02.007

20 Huebner GM, Hamilton I, Chalabi Z, *et al.* Comparison of indoor temperatures of homes with recommended temperatures and effects of disability and age: an observational, cross-sectional study. *BMJ Open* 2018;**8**:e021085. doi:10.1136/bmjopen-2017-021085

21 Lelkes O, Zolyomi E. Quality of housing and the link to income. Brussels: : European Commission 2009.

22 Tunstall R, Bevan M, Bradshaw J, *et al.* The links between housing and poverty: an evidence review. York: : Joseph Rowntree Foundation 2013.

23 Marshall C, Moody A, Hamilton I, *et al.* Changes in heating of homes from 1991 to 2011 using data from the ONS-Longitudinal Study.

24 Ministry of Housing, Communities and Local Government. English Housing Survey 2016 to 2017: headline report. London: : MHCLG 2018.

25 Oreszczyn T, Hong SH, Ridley I, *et al.* Determinants of winter indoor temperatures in low income households in England. *Energy and Buildings* 2006;**38**:245–52. doi:10.1016/j.enbuild.2005.06.006

26 Public Health England. PHE heatwave mortality monitoring, Summer 2019. London: : PHE https://assets.publishing.service.gov.uk/government/uploads/system/uploads/attachment_data/file/841320/PHE_heatwave_report_2019.pdf (accessed 24 Jul 2020).

27 Hajat S, Gasparrini A. The Excess Winter Deaths Measure: Why Its Use Is Misleading for Public Health Understanding of Cold-related Health Impacts. *Epidemiology* 2016;**27**:486–91. doi:10.1097/EDE.0000000000000479

28 Mindell J, Biddulph JP, Hirani V, *et al.* Cohort Profile: The Health Survey for England. *International Journal of Epidemiology* 2012;**41**:1585–93. doi:10.1093/ije/dyr199

29 Mindell JS, Goesswald A, Kamtsiuris P, *et al.* Sample selection, recruitment and participation rates in health examination surveys in Europe – experience from seven national surveys. *BMC Med Res Methodol* 2015;**15**:78. doi:10.1186/s12874-015-0072-4

For peer review only

For peer review only - http://bmjopen.bmj.com/site/about/guidelines.xhtml

30 Craig R, Fuller E, Mindell J (Eds.). Health Survey for England 2014. Volume 2. Methods and documentation. Leeds: : NHS Digital 2015. https://digital.nhs.uk/data-and-information/publications/statistical/health-survey-for-england/health-survey-for-england-2014

31 de Bruin A, Picavet HSJ, Nossikov A, editors. *Health interview surveys: towards international harmonization of methods and instruments*. Copenhagen: : World Health Organization, Regional Office for Europe 1996. https://www.euro.who.int/__data/assets/pdf_file/0017/111149/E72841.pdf?ua=1 (accessed 14 Jul 2020).

32 Nixon O. General health harmonised principle. Government Statistical Service. 2020.https://gss.civilservice.gov.uk/policy-store/general-health/ (accessed 14 Jul 2020).

33 The National Statistics Socio-economic classification (NS-SEC). Office for National Statistics. 2016.https://www.ons.gov.uk/methodology/classificationsandstandards/otherclassifications/thenationalstatisticssocioeconomicclassificationnssecrebasedonsoc2010 (accessed 15 Jul 2020).

34 Pollard A, Jones T, Sherratt S, *et al.* Use of Simple Telemetry to Reduce the Health Impacts of Fuel Poverty and Living in Cold Homes. *Int J Environ Res Public Health* 2019;**16**. doi:10.3390/ijerph16162853

35 Park J. One Hundred Years of Housing space Standards. what now?Housing Space Standards. London: : Levitt Bernstein 2017. https://levittbernstein.co.uk/site/assets/files/2682/one_hundred_years_of_space_standards.pdf

36 Nakajima Y, Schmidt SM, Malmgren Fänge A, *et al.* Relationship between Perceived Indoor Temperature and Self-Reported Risk for Frailty among Community-Dwelling Older People. *Int J Environ Res Public Health* 2019;**16**. doi:10.3390/ijerph16040613

37 Armstrong B, Bonnington O, Chalabi Z, *et al. The impact of home energy efficiency interventions and winter fuel payments on winter- and cold-related mortality and morbidity in England: a natural equipment mixed-methods study*. Southampton (UK): : NIHR Journals Library 2018. http://www.ncbi.nlm.nih.gov/books/NBK532137/ (accessed 3 Dec 2019).

38 Huebner GM, McMichael M, Shipworth D, *et al.* The shape of warmth: temperature profiles in living rooms. *Building Research & Information* 2015;**43**:185–96. doi:10.1080/09613218.2014.922339

39 Simon JG, De Boer JB, Joung IMA, *et al.* How is your health in general? A qualitative study on self-assessed health. *European Journal of Public Health* 2005;**15**:200–8. doi:10.1093/eurpub/cki102

40 Jylhä M. What is self-rated health and why does it predict mortality? Towards a unified conceptual model. *Social Science & Medicine* 2009;**69**:307–16. doi:10.1016/j.socscimed.2009.05.013

41 Heistaro S, Jousilahti P, Lahelma E, *et al.* Self rated health and mortality: a long term prospective study in eastern Finland. *Journal of Epidemiology & Community Health* 2001;**55**:227–32. doi:10.1136/jech.55.4.227

For peer review only

For peer review only - http://bmjopen.bmj.com/site/about/guidelines.xhtml

42 Idler EL, Benyamini Y. Self-rated health and mortality: a review of twenty-seven community studies. *J Health Soc Behav* 1997;**38**:21–37.

43 Kaplan GA, Camacho T. Perceived health and mortality: A nine-year follow-up of the Human Population Laboratory Cohort. *American Journal of Epidemiology* 1983;**117**:292–304. doi:10.1093/oxfordjournals.aje.a113541

44 Møller L, Kristensen TS, Hollnagel H. Self rated health as a predictor of coronary heart disease in Copenhagen, Denmark. *Journal of Epidemiology & Community Health* 1996;**50**:423–8. doi:10.1136/jech.50.4.423

45 Mossey JM, Shapiro E. Self-rated health: a predictor of mortality among the elderly. *Am J Public Health* 1982;**72**:800–8. doi:10.2105/AJPH.72.8.800

46 Benjamins MR, Hummer RA, Eberstein IW, *et al.* Self-reported health and adult mortality risk: An analysis of cause-specific mortality. *Social Science & Medicine* 2004;**59**:1297–306. doi:10.1016/j.socscimed.2003.01.001

47 Wannamethee G, Shaper AG. Self-assessment of Health Status and Mortality in Middle-Aged British Men. *Int J Epidemiol* 1991;**20**:239–45. doi:10.1093/ije/20.1.239

48 Idler EL, Kasl SV. Self-Ratings of Health: Do they also Predict change in Functional Ability? *The Journals of Gerontology Series B: Psychological Sciences and Social Sciences* 1995;**50B**:S344–53. doi:10.1093/geronb/50B.6.S344

49 Singh-Manoux A. What does self rated health measure? Results from the British Whitehall II and French Gazel cohort studies. *Journal of Epidemiology & Community Health* 2006;**60**:364–72. doi:10.1136/jech.2005.039883

50 Umishio W, Ikaga T, Kario K, *et al.* Cross-Sectional Analysis of the Relationship Between Home Blood Pressure and Indoor Temperature in Winter: A Nationwide Smart Wellness Housing Survey in Japan. *Hypertension* 2019;**74**:756–66. doi:10.1161/HYPERTENSIONAHA.119.12914

51 Wang Q, Li C, Guo Y, *et al.* Environmental ambient temperature and blood pressure in adults: A systematic review and meta-analysis. *Science of The Total Environment* 2017;**575**:276–86. doi:10.1016/j.scitotenv.2016.10.019

52 Zhao H, Jivraj S, Moody A. “My blood pressure is low today, do you have the heating on?” The association between indoor temperature and blood pressure. *J Hypertens* 2019;**37**:504–12. doi:10.1097/HJH.0000000000001924

53 Cabrera SE, Mindell JS, Toledo M, *et al.* Associations of Blood Pressure With Geographical Latitude, Solar Radiation, and Ambient Temperature: Results From the Chilean Health Survey, 2009–2010: Table 1. *Am J Epidemiol* 2016;**183**:1071–3. doi:10.1093/aje/kww037

54 World Health Organization Regional Office for Europe. Housing, energy and thermal comfort : a review of 10 countries within the WHO European Region. Copenhagen: : WHO Regional Office for Europe 2007. https://apps.who.int/iris/handle/10665/107815

55 World Health Organization. The Effects of the Indoor Housing Climate on the Health of the Elderly: Report on a WHO Working Group, Graz, Austria, 20-24 September 1982. Copenhagen: : WHO Regional Office for Europe 1984.

For peer review only - http://bmjopen.bmj.com/site/about/guidelines.xhtml

56 Bone D, Carmichael K, Wookey R. The Cold Weather Plan for England: Protecting health and reducing harm from cold weather. London: : Public Health England 2014.

57 Wookey R, Bone A, Carmichael C, *et al.* Minimum home temperature thresholds for health in winter – A systematic literature review. London: : Public Health England 2014.

58 Angelini V, Daly M, Moro M, *et al. The effect of the Winter Fuel Payment on household temperature and health: a regression discontinuity design study*. Southampton (UK): : NIHR Journals Library 2019. http://www.ncbi.nlm.nih.gov/books/NBK535694/ (accessed 3 Dec 2019).

For peer review only

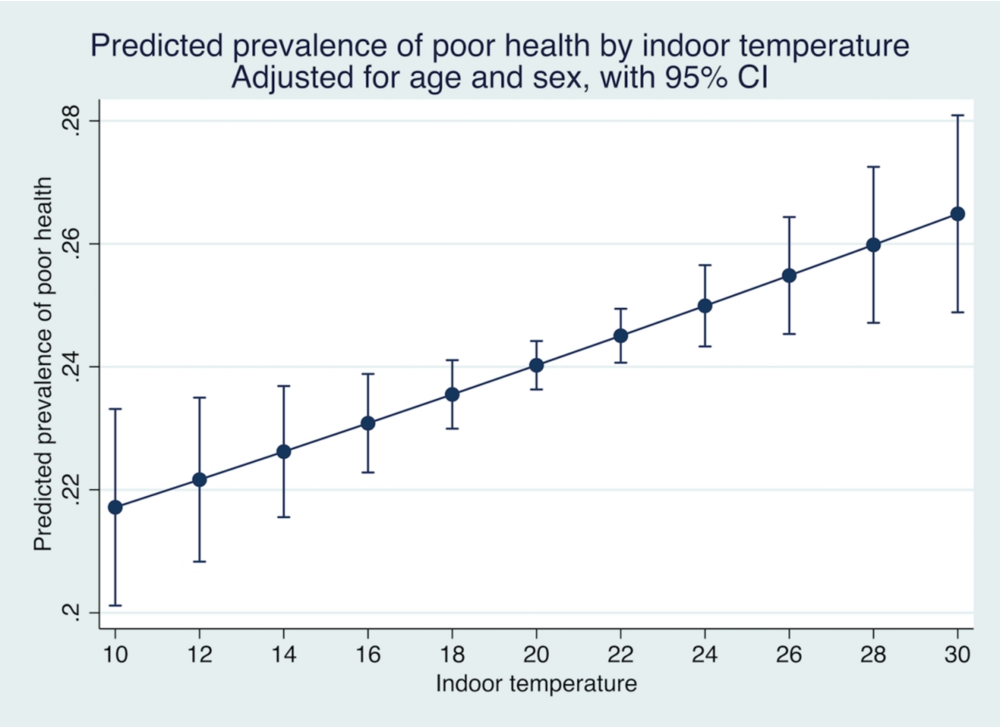

Caption: Predicted prevalence of poor health for each degree increase in indoor temperature
Legend: Prevalence of poor health predicted by the logistic regression of indoor temperature on poor health, adjusted for age and sex. OR 1.01 (95% CI 1.00-1.02), see Table 3, model 1.

123x90mm (300 x 300 DPI)

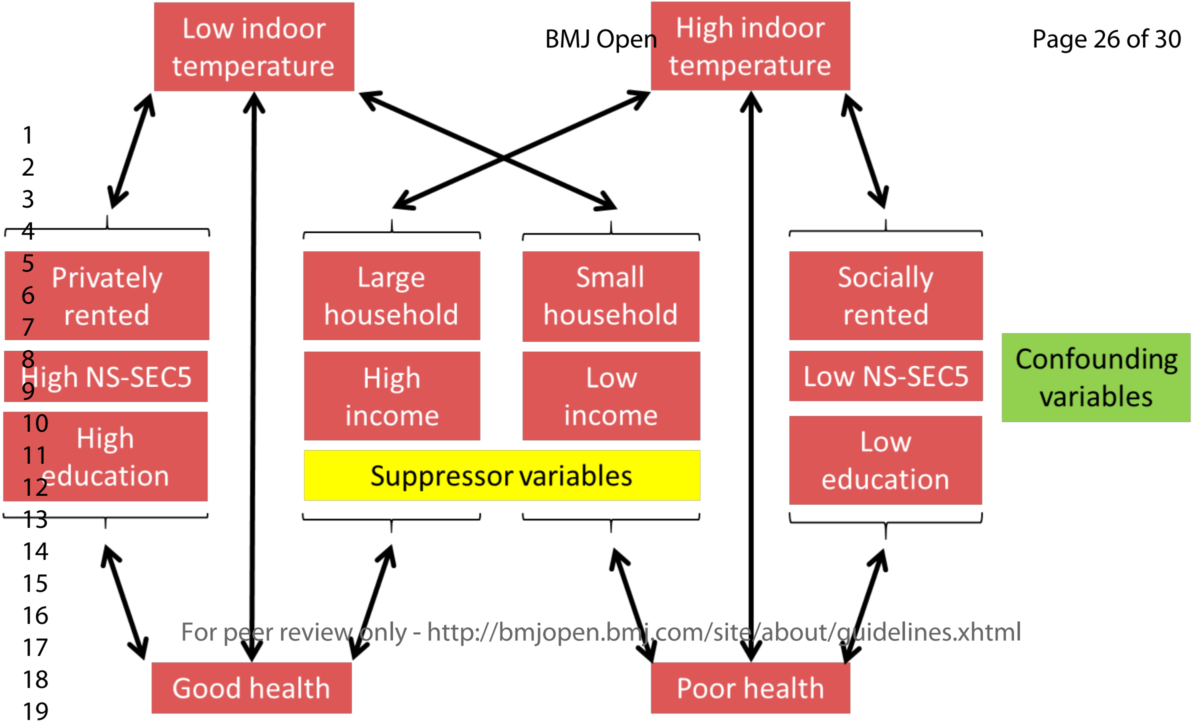

For peer review only - http://bmjopen.bmj.com/site/about/guidelines.xhtml

Figure S1. Flow diagram showing selection of data into analytical sample

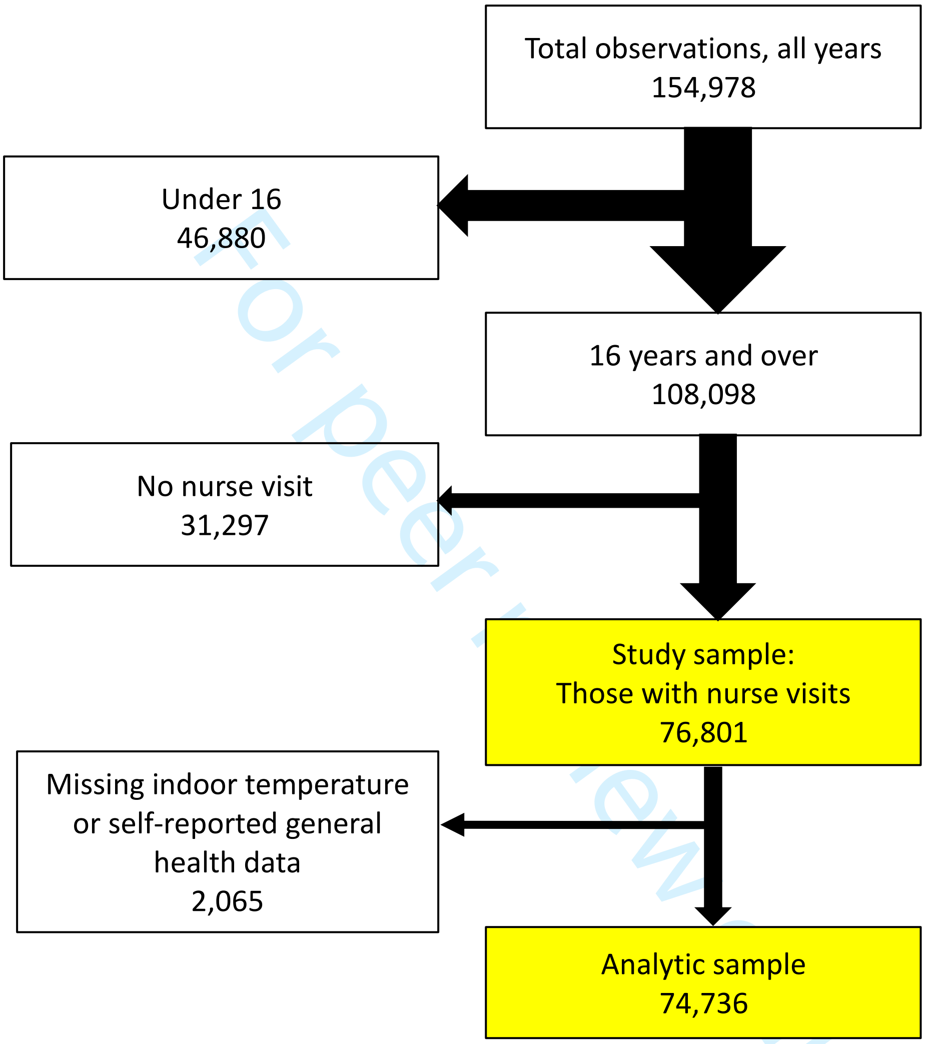

For peer review only

Supplementary material

Table S1. Characteristics of participants

Variable	Category	Analytical sample[a] (N=74,736)		Excluded from analysis[b] (N=2065)		Study Sample[c] (N=76,801)	
		N*	%	N	%	N	%
Gender[d]	Men	33,526	45%	533	26%	34,059	44%
	Women	41,210	55%	1,532	74%	42,742	56%
Age group[d]	16-24	6,593	9%	373	18%	6,966	9%
	25-34	9,589	13%	639	31%	10,228	13%
	35-44	13,145	18%	375	18%	13,520	18%
	45-54	12,929	17%	179	9%	13,108	17%
	55-64	12,753	17%	161	8%	12,914	17%
	65-74	11,315	15%	162	8%	11,477	15%
	75+	8,412	11%	176	9%	8,588	11%
NS-SEC5[d]	Managerial/ professional	25,297	34%	557	27%	25,854	34%
	Intermediate Occupation	10,354	14%	234	11%	10,588	14%
	Small employers	6,514	9%	91	4%	6,605	9%
	Lower supervisory	6,087	8%	96	5%	6,183	8%
	Semi-routine	23,314	31%	511	25%	23,825	31%
	Missing data	*3,170*	*4%*	132	6%	*3,302*	*4%*
Education[d]	Degree level or higher	15,400	21%	476	23%	15,876	21%
	Qualification below degree	40,233	54%	992	48%	41,225	54%
	No qualification	19,103	26%	515	25%	19,618	26%
	Missing data	*0*	*0%*	*82*	*4%*	*82*	*0.1%*
Income quintile[d]	Lowest	10,861	15%	420	20%	11,281	15%
	2nd lowest	12,115	16%	302	15%	12,417	16%
	3rd	13,190	18%	260	13%	13,450	18%
	4th	13,537	18%	265	13%	13,802	18%
	Highest	13,291	18%	321	16%	13,612	18%
	Missing data	*11,742*	*16%*	461	22%	*12,203*	*16%*
Tenure[d]	Owner-occupied	55,159	74%	1,076	52%	56,235	73%
	Privately rented	6,476	9%	283	14%	6,759	9%
	Socially rented	11,347	15%	492	24%	11,839	15%
	Missing data	*1,754*	*2%*	214	10%	*1,968*	*3%*
Household size[d]	1	13,677	18%	284	14%	13,961	18%
	2	30,117	40%	712	34%	30,829	40%
	3	12,585	17%	540	26%	13,125	17%
	4	12,173	16%	323	16%	12,496	16%
	5+	6,184	8%	206	10%	6,390	8%

For peer review only

Tertile of regional mean outdoor temperature at month of visit[e]	Low	24,927	33%	653	32%	25,580	33%
	Medium	24,506	33%	752	35%	25,238	33%
	High	25,303	34%	680	33%	25,983	34%
General health[f]	Good	55,235	74%	1,567	76%	56,802	74%
	Poor	19,501	26%	498	24%	19,999	26%
Longstanding illness[d]	No	39,246	53%	1,269	61%	40,515	53%
	Yes	35,490	47%	781	38%	36,271	47%
Heart condition[d]	No	63,905	86%	1,838	89%	65,743	86%
	Yes	10,831	14%	212	10%	11,043	14%
Respiratory condition[g]	No	68,051	91%	1,869	91%	69,920	91%
	Yes	6,685	9%	181	9%	6,866	9%
Total		*74,736*	*97.3%*	*2,065*	*2.7%*	*76,801*	*100%*

[a] Analytical sample was all participants with a nurse visit and with information on both self-rated health and indoor temperature'.

[b] Excluded sample are those HSE participants who had a nurse visit (so are included in the study sample) but had missing data for indoor temperature and/or self-rated health

[c] Study sample: all those with a nurse visit

[d] X^2 test for difference between analytic sample and excluded participants: p<0.001

[e] X^2 test for difference between analytic sample and excluded participants: p=0.036

[f] X^2 test for difference between analytic sample and excluded participants: p=0.043

[g] X^2 test for difference between analytic sample and excluded participants: p=0.858

For peer review only

STROBE Statement—Checklist of items that should be included in reports of ***cross-sectional studies***

	Item No	Recommendation	This manuscript	Line no.[1]
**Title and abstract**	1	(*a*) Indicate the study's design with a commonly used term in the title or the abstract	The word 'cross-sectional' in the title; 'cross-sectional study' & 'annual … survey' in the abstract	2; 48; 53-54
		(*b*) Provide in the abstract an informative and balanced summary of what was done and what was found	Yes	52-65
**Introduction**				
Background / rationale	2	Explain the scientific background and rationale for the investigation being reported	Yes	79-128
Objectives	3	State specific objectives, including any prespecified hypotheses	Yes	129-134
**Methods**				
Study design	4	Present key elements of study design early in the paper	Yes	138-151
Setting	5	Describe the setting, locations, and relevant dates, including periods of recruitment, exposure, follow-up, and data collection	Yes	138-151; 186-238
Participants	6	(*a*) Give the eligibility criteria, and the sources and methods of selection of participants	Yes	153-178
Variables	7	Clearly define all outcomes, exposures, predictors, potential confounders, and effect modifiers. Give diagnostic criteria, if applicable	Yes	186-238
Data sources / measurement	8*	For each variable of interest, give sources of data and details of methods of assessment (measurement). Describe comparability of assessment methods if there is more than one group	Yes	186-238
Bias	9	Describe any efforts to address potential sources of bias	Yes	278-284; 387-390
Study size	10	Explain how the study size was arrived at	Yes	134-140; 147-150
Quantitative variables	11	Explain how quantitative variables were handled in the analyses. If applicable, describe which groupings were chosen and why	Yes	187-223;
Statistical methods	12	(*a*) Describe all statistical methods, including those used to control for confounding	Yes	245-284
		(*b*) Describe any methods used to examine subgroups and interactions	Yes	270-277
		(*c*) Explain how missing data were addressed	Yes	245-250
		(*d*) If applicable, describe analytical methods taking account of sampling strategy	Yes	278-284
		(*e*) Describe any sensitivity analyses	Yes	272-274
**Results**				
Participants	13*	(a) Report numbers of individuals at each stage of study—eg numbers potentially eligible, examined for eligibility, confirmed eligible, included in the study, completing follow-up, and analysed	Yes	166-178; Fig S1
		(b) Give reasons for non-participation at each stage	Yes	Fig S1
		(c) Consider use of a flow diagram	Yes	Fig S1

[1] Line numbers refer to the version with tracked changes

For peer review only - http://bmjopen.bmj.com/site/about/guidelines.xhtml

Descriptive data	14*	(a) Give characteristics of study participants (eg demographic, clinical, social) and information on exposures and potential confounders	Yes	Tab S1
		(b) Indicate number of participants with missing data for each variable of interest	N/A	147-148
Outcome data	15*	Report numbers of outcome events or summary measures	N/A	
Main results	16	(*a*) Give unadjusted estimates and, if applicable, confounder-adjusted estimates and their precision (eg, 95% confidence interval). Make clear which confounders were adjusted for and why they were included	95% CIs provided for minimally-adjusted (age and outdoor temperature) & fully adjusted models	Tab 1; Tab 2; Tab 3
		(*b*) Report category boundaries when continuous variables were categorized	Yes (age-group; tertiles of temperatures)	254-255; 271-274
		(*c*) If relevant, consider translating estimates of relative risk into absolute risk for a meaningful time period	N/A	
Other analyses	17	Report other analyses done—eg analyses of subgroups and interactions, and sensitivity analyses	Tertiles of indoor & outdoor temp	337-341; 345-348
**Discussion**				
Key results	18	Summarise key results with reference to study objectives	Yes	356-361
Limitations	19	Discuss limitations of the study, taking into account sources of potential bias or imprecision. Discuss both direction and magnitude of any potential bias	Yes	76-77; 392-403
Interpretation	20	Give a cautious overall interpretation of results considering objectives, limitations, multiplicity of analyses, results from similar studies, and other relevant evidence	Yes	361-373
Generalisability	21	Discuss the generalisability (external validity) of the study results	Yes	386-391
**Other information**				
Funding	22	Give the source of funding and the role of the funders for the present study and, if applicable, for the original study on which the present article is based	Yes	479-484

*Give information separately for exposed and unexposed groups.

Note: An Explanation and Elaboration article discusses each checklist item and gives methodological background and published examples of transparent reporting. The STROBE checklist is best used in conjunction with this article (freely available on the Web sites of PLoS Medicine at http://www.plosmedicine.org/, Annals of Internal Medicine at http://www.annals.org/, and Epidemiology at http://www.epidem.com/). Information on the STROBE Initiative is available at www.strobe-statement.org.

For peer review only
